# Point nodes persisting far beyond *T*_c_ in Bi2212

**DOI:** 10.1038/ncomms8699

**Published:** 2015-07-09

**Authors:** Takeshi Kondo, W. Malaeb, Y. Ishida, T. Sasagawa, H. Sakamoto, Tsunehiro Takeuchi, T. Tohyama, S. Shin

**Affiliations:** 1 ISSP, University of Tokyo, Kashiwa, Chiba 277-8581, Japan; 2 Materials and Structures Laboratory, Tokyo Institute of Technology, Yokohama, Kanagawa 226-8503, Japan; 3 Department of Crystalline Materials Science, Nagoya University, Nagoya 464-8603, Japan; 4 Energy Materials Laboratory, Toyota Technological Institute, Nagoya 468-8511, Japan; 5 Department of Applied Physics, Tokyo University of Science, Tokyo 125-8585, Japan

## Abstract

In contrast to a complex feature of antinodal state, suffering from competing orders, the pairing gap of cuprates is obtained in the nodal region, which therefore holds the key to the superconducting mechanism. One of the biggest question is whether the point nodal state as a hallmark of *d*-wave pairing collapses at *T*_c_ like the BCS-type superconductors, or it instead survives above *T*_c_ turning into the preformed pair state. A difficulty in this issue comes from the small magnitude of the nodal gap, which has been preventing experimentalists from solving it. Here we use a laser ARPES capable of ultrahigh-energy resolution, and detect the point nodes surviving far beyond *T*_c_ in Bi2212. By tracking the temperature evolution of spectra, we reveal that the superconductivity occurs when the pair-breaking rate is suppressed smaller than the single-particle scattering rate on cooling, which governs the value of *T*_c_ in cuprates.

## Abstract (web summary)

The pairing gap of the high-*T*_c_ cuprates has been expected to close at the transition temperature, similarly to the case of conventional superconductors. Here the authors perform ARPES measurements on Bi2212, and reveal a point nodal gap formation beyond *T*_c_, characterized in terms of three parameters.

## Graphical abstract

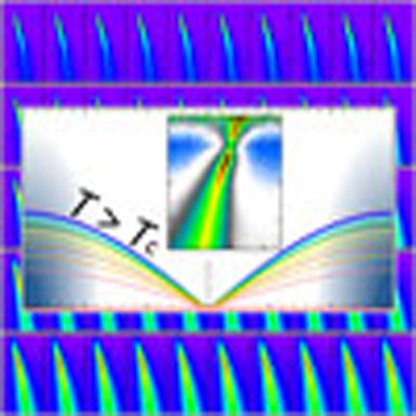

In cuprates, the energy gap (pseudogap) starts opening at a temperature much higher than *T*_c_, in some cases above room temperature. Many experimental evidences^1^,^2^,^3^,^4^,^5^,^6^,^7^,^8^,^9^ point to a competing-order origin, rather than the preformed pair, for the pseudogap observed around the antinode with the maximum energy gap. The pseudogap state has been revealed to sharply diminishes toward the antinode and disappear far off the node by the spectral weight analysis for the angle-resolved photoemission spectroscopy (ARPES) data of optimally doped samples^9^. This is compatible with the recent results by the scanning tunnelling spectroscopy (STM) and the resonant X-ray scattering, showing that the density wave Q-vectors are detected only in the Cu–O bond directions^5^,^7^,^10^,^11^,^12^. While the whole Fermi surface is eventually dominated by the pseudogap close to the Mott insulating phase^6^,^13^,^14^, the ‘pure' pairing state seems to be realized around the node at least in the optimally and overdoped regime^9^,^15^,^16^,^17^,^18^,^19^,^20^,^21^. The relevant feature to it would be the spatially homogeneous electronic state seen at the low bias in the STM spectra, which reflects the nodal momentum region^11^,^20^,^22^. It strongly contrasts to the highly inhomogeneous spectra at the high bias associated with the antinodal states. Unveiling the nature of the spectral gap near the node is therefore crucial to elucidate the superconducting mechanism in cuprates. A difficulty however is the small magnitude of the gap, which has been challenging the experimentalists to investigate.

It has been proposed that the pairing-gap evolution with temperature simply follows the conventional Bardeen-Cooper-Schrieffer (BCS) function^15^, and Fermi arcs (disconnected segments of gapless Fermi surface)^23^ emerge at *T*_c_^6^,^15^,^16^,^17^,^20^,^21^,^22^,^23^,^24^, marking momentum borders between the superconducting and the competing pseudogap regions^6^,^15^. However, it seems contradicting the observations of the Nernst and diamagnetic effects above *T*_c_^25^,^26^, which are viewed as signatures of a phase-incoherent superconductivity.

Recently, a contrasting view was proposed^9^,^18^,^19^: its underlying idea is that one should discard the notion of electron quasiparticles, instead pay attention to the density of states, which is an effective way of judging the existence of energy gap. Accordingly, a momentum integration of ARPES spectra has been performed over a selected part of the momentum space. This quantity contributed from the nodal region was found to have a gap-like structure even above *T*_c_^18^,^19^. The result seems to be in direct opposition to the above widely accepted view. Nevertheless, the evidence for single-particle gap with the point nodes surviving above *T*_c_ is still missing, and strongly desired to unveil the nature of Fermi arc, which is tied to the pairing mechanism of cuprates.

The determination of the momentum-resolved gap structure has been also attempted by STM studies through the spectral line-shape analysis^20^ and by applying the octet model to the interference pattern^21^,^27^,^28^. While these STM techniques are very successful, they seem to be limited to the investigation of the antinodal region; the gap structure close to the node is not determined^27^,^28^, or the gapless Fermi arc is obtained even below *T*_c_^20^,^21^, which has never been reported by the ARPES studies. It is thus crucial to investigate the nodal region by means of the ARPES, which is specialized for an observation of the momentum space.

Here we examine the momentum-resolved single-particle spectra of Bi2212 obtained by a laser ARPES^29^. The ultrahigh-energy resolution and bulk sensitivity achieved by utilizing a low-energy laser source (*hν*=7 eV) enabled us to obtain high-quality spectra with an extremely sharp line shape. We demonstrate, within the quasiparticle picture, the absence of the gapless Fermi arc at *T*_c_, and an isotropic temperature evolution of point nodal pairing state persistent far above *T*_c_. We find that not only the single-particle scattering rate (Γ_single_), but also the pair-breaking rate (Γ_pair_) is required to reproduce the ARPES spectra. Furthermore, the magnitude of *T*_c_ is determined by the mutual relation between Γ_single_ and Γ_pair_ in the form of Γ_single_(*T*_c_)=Γ_pair_(*T*_c_). Importantly, the momentum-integrated spectra of ARPES and STM previously investigated are not capable of separating these two quantities (Γ_single_ and Γ_pair_) (see Supplementary Note 4); thus, the present results provide novel ingredients essential to formulate the pairing mechanism of cuperates.

## Results

### Absence of Fermi arc at *T*_c_

**Figure 1:**
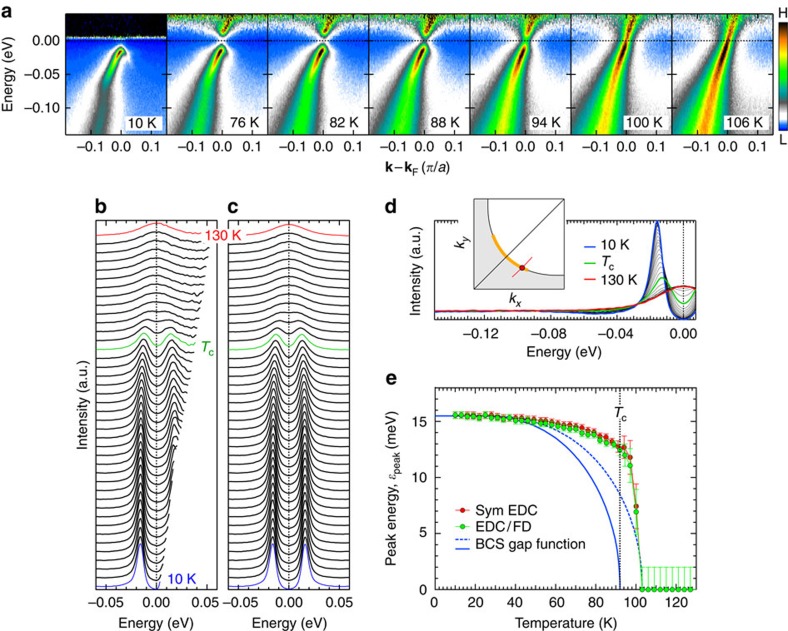
Temperature evolution of ARPES spectra in the nodal region. (**a**) Dispersion maps at several temperatures measured along a momentum cut close to the node in OP92K (a red line in the inset of **d**). Each map is divided by the Fermi function at the measured temperature. (**b**) Temperate evolution of EDCs at **k**_F_ (a circle in the inset of **d**) from deep below (10 K) to much higher than *T*_c_ (130 K). Each spectrum is divided by the Fermi function at the measured temperature. (**c**) The same data as in **b**, but symmetrized about *E*_F_. (**d**) The same data as in **c** plotted without an offset. The inset represents the Fermi surface. The bold orange line indicates the momentum region where the Fermi arc was previously claimed to emerge at *T*_c_. (**e**) Peak energies of spectra in **b** and **c** plotted as a function of temperature, *ɛ*_peak_. The solid and dashed blue curves show the BCS gap function with an onset at *T*_c_ (92 K) and slightly above *T*_c_, respectively. Error bars in **e** represent standard deviations of the spectral peak positions.

In Fig. 1, we show typical data obtained inside the nodal region where the Fermi arc (bold orange curve in the inset of Fig. 1d) was previously claimed to appear at *T*_c_^6^,^15^,^16^,^20^,^24^. The ARPES intensity map divided by the Fermi function (see Fig. 1a) shows an energy gap and an upper branch of the Bogoliubov dispersion at low temperatures, as an indication of the pairing state. We extract the spectra at the Fermi momentum (**k**_F_) over a wide range of temperature in Fig. 1b, and plot the peak energies (*ɛ*_peak_) in Fig. 1e. In the same panel, we also plot *ɛ*_peak_ of energy distribution curves (EDCs) symmetrized about the Fermi energy (*E*_F_) to remove the effect of Fermi cutoff^23^, and confirm a consistency between the two different methods. Our high-quality data clearly exhibit that the gap is open even at *T*_c_ (=92 K) (see green spectra in Fig. 1b,c), and the *ɛ*_peak_(*T*) disagrees with the BCS gap evolution (blue solid curve in Fig. 1e). Even if assuming a phase fluctuation slightly above *T*_c_, the BCS-type curve (blue dashed curve) still does not fit to the data.

**Figure 2:**
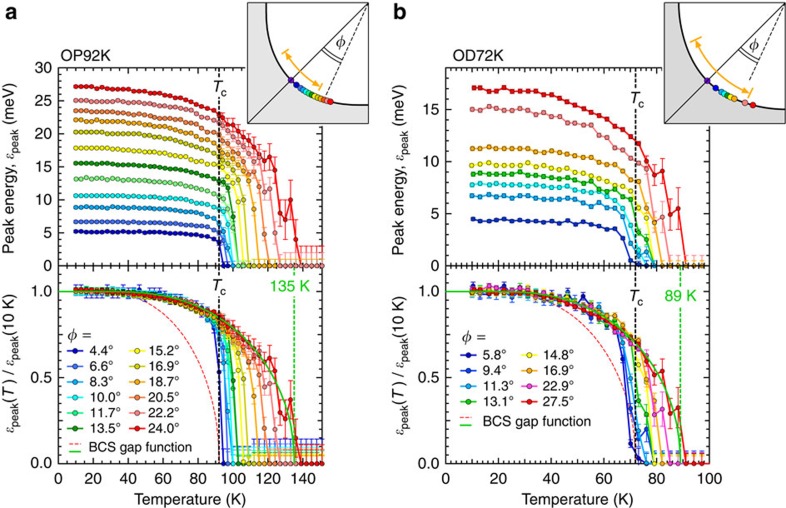
Disagreement between the ARPES results and the conventional BCS gap function. (**a**,**b**) Temperature dependence of spectral peak energy, *ɛ*_peak_(*T*), at various **k**_F_ points (colour circles in the insets) for OP92K and OD72K, respectively. In the bottom of each panel, the same curves of *ɛ*_peak_(*T*) are normalized to the maximum value at the lowest temperature. A red dashed curve and a green solid curve are the BCS gap function with the onset at *T*_c_ and *T*_pair_, respectively (*T*_pair_=135 K for OP92K and *T*_pair_=89 K for OD89K). The inset shows the Fermi surface with measured **k**_F_ points (colour circles). The bold orange line indicates the momentum region where the Fermi arc was previously claimed to emerge at *T*_c_. Error bars in **a** and **b** represent standard deviations of the spectral peak positions.

**Figure 3:**
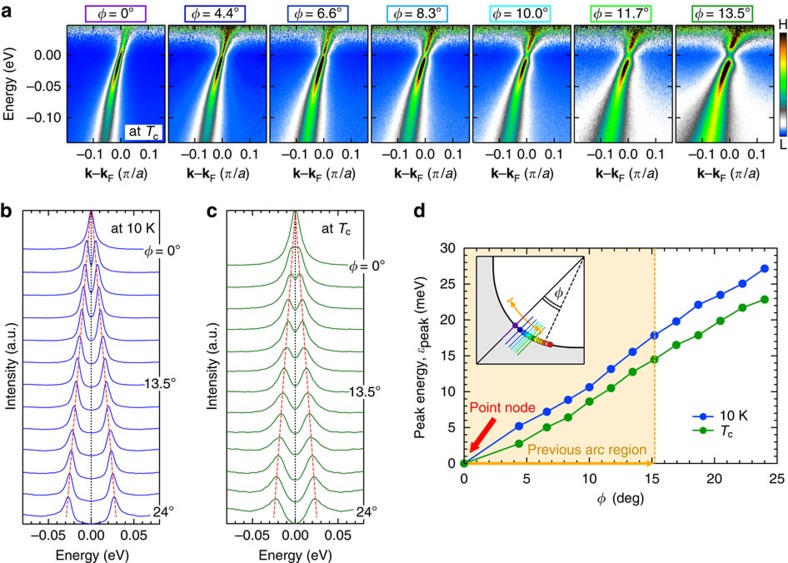
Momentum variation of spectra showing the absence of Fermi arc at *T*_c_. (**a**) Dispersion maps at *T*_c_ along several momentum cuts (colour lines in the inset of **d** measured for OP92K. Each map is divided by the Fermi function at the measured temperature. The described *φ* are the directions of measured **k**_F_ points (*φ* is defined in the inset of **d**). (**b**,**c**) Symmetrized EDCs at **k**_F_ over a wide range of angle *φ* (colour circles in the inset of **d**) at 10 K and *T*_c_ (=92 K), respectively. The red dotted lines are guide to the eyes for the gap evolution. (**d**) Fermi angle *φ* dependence of peak energies of spectra in **b** (*ɛ*_peak_(10 K)) and **c** (*ɛ*_peak_(*T*_c_)). The orange arrows indicate the momentum region where the Fermi arc was previously claimed to emerge at *T*_c_.

To pin down the cause of this anomaly, we examine the momentum variation of *ɛ*_peak_(*T*) for the optimally and overdoped samples (OP92K and OD72K) in Fig. 2a,b, respectively. Surprisingly, the gap does not close at *T*_c_ regardless of **k**_F_ points. The symmetrized EDCs around the node for OP92K are plotted in Fig. 3b (*T*=10 K) and Fig. 3c (*T*=*T*_c_). We find that the *d*-wave gap with a point node persists at *T*_c_ (Fig. 3d); the Fermi arc is absent. While a small uncertainty in gap estimation remains in the close vicinity of the node due to the finite spectral width, it is negligible compared with the previously reported extensive gapless Fermi arc (orange arrows in the inset of Fig. 2a,b). The absence of Fermi arc is further confirmed in Fermi function divided band dispersions (Fig. 3a) measured at *T*_c_ along several momentum cuts (colour lines in the inset of Fig. 3d). The loss of spectral weight at *E*_F_ due to the gap opening is seen in all the maps except for at the node (see Supplementary Fig. 6 for more details). Our high-resolution data also show other inconsistencies with the previous expectations^6^,^15^,^16^,^17^. First, the length of arc with single spectral peaks (*ɛ*_peak_=0) is not linearly extrapolated to zero at *T*=0 against the nodal liquid behaviour (Supplementary Fig. 7)^16^,^17^. Second, the temperature evolution of such an arc is gradual up to far above *T*_c_ with no indication of momentum borders separating two distinct states^6^,^15^.

For a further examination, we normalize each curve of *ɛ*_peak_(*T*) to the maximum value at the lowest temperature in the bottom panels of Fig. 2a (OP92K) and 2b (OD72K). One can confirm that the data are mismatched with the conventional BCS curve (red dashed curves) even in the close vicinity of the node. More importantly, the *ɛ*_peak_(*T*) behaviour with a steep drop to zero becomes more gradual with getting away from the node, and it eventually follows a BCS-type gap function (green curves) with an onset much higher than *T*_c_ (∼135 K and ∼89 K for OP92K and OD72K, respectively).

### Point nodal gap above *T*_c_ by the leading edge shift

**Figure 4:**
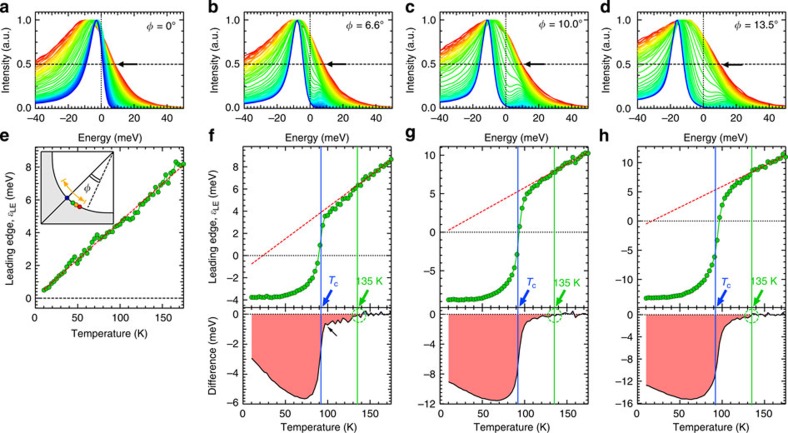
Evidence for the point nodal gap surviving above *T*_c_ revealed by means of the energy shift of spectral leading edge. (**a**) The temperature evolution of ARPES spectra (EDCs) at the gap node for OP92K, normalized to the peak intensity of each curve. (**b**–**d**) The same spectra as **a**, but measured off the node (colour circles in the inset of **e**). (**e**–**h**) Temperature dependence of the spectral leading edge, *ɛ*_LE_(*T*), defined as energies where the spectral peaks become half in intensity (marked by arrows in **a**–**d**). The bottom panels of **f**–**h** plot the difference of *ɛ*_LE_(*T*) from the *T*-linear behaviours (red dashed lines), which are fit to *ɛ*_LE_(*T*) at high temperatures. The onset temperatures of the deviation from *T*-linear behaviour are around 135 K for all the **k**_F_ points off the node (dashed circles). A kink is seen in the difference curve for **f** (small black arrow) because the thermally populated Bogoliubov peaks in the unoccupied side significantly affect the shapes of leading edge specially at **k**_F_s close to the node with small gaps. The inset of **e** shows the Fermi surface with the examined **k**_F_ points. The bold orange arrow indicates the momentum region where the Fermi arc was previously claimed to emerge at *T*_c_.

To clarify the anomalous feature above *T*_c_, here we investigate another spectral measure, so-called leading edge of EDC, which is also commonly used for a gap estimation. Figure 4a–d shows the non-symmetrized EDCs of OP92K, normalized to the peak intensity of each curve. The energy location of spectral leading edge (*ɛ*_LE_), at which the spectral intensity becomes half (allows in **a**–**d**), is plotted in Fig. 4e–h as a function of temperature. The nodal values (Fig. 4e) have a *T*-linear behaviour, which is expected for the spectra dominated by the Fermi cutoff effect. While the nodal *ɛ*_LE_(*T*) could have a complex behaviour^30^ with a poor energy resolution, such a effect seems not to be observed in our case. Hence, even the slightest energy gap could be detected as the deviation of *ɛ*_LE_(*T*) from the *T*-linear behaviour. Such a deviation is indeed observed for all the **k** points off the node (Fig. 4f–h). It is more clearly demonstrated in the bottom panels of Fig. 4f–h, where the difference of *ɛ*_LE_(*T*) from the *T*-linear behaviour is extracted. An astonishing result we found is that the onset temperatures are almost the same (∼135 K) regardless of the directions (*φ*), which validates that the point nodal state persists up to ∼135 K.

Importantly, the gap opening temperature is coincident with the onset of the BCS-type gap function obtained with the symmetrized EDCs far off the node (a green curve in Fig. 2a). A plausible explanation for it is that the spectral peak energy underestimates the ‘real' energy gap (*ɛ*_peak_<Δ)^9^,^31^,^32^, and energy gaps (Δ) comparable to or smaller than the peak width cannot be detected by tracing the peak positions (see a simulation in the Supplementary Fig. 8). This situation could occur at high temperatures, and it gets more serious towards the node with smaller Δ. We actually detect the signature of such a small gap in the off-nodal spectra with a single peak above *T*_c_ (Supplementary Fig. 10); the spectral width becomes smaller with increasing temperature up to ∼135 K, which contrasts to the monotonic broadening seen in the nodal direction. The characteristic momentum variation of *ɛ*_peak_(*T*) in Fig. 2a,b, therefore, could be a natural consequence of Δ(*T*) having the same onset temperature at ∼135 K and ∼89 K, respectively, for all directions (*φ*).

### Anomalous pair formation in cuprates

We find below that a model spectral function, *πA*(**k**_F_, *ω*)=Σ′′/[(*ω*−Σ′)^2^+Σ′′^2^], with such a BCS-type Δ(*T*, *φ*) indeed reproduces the ARPES spectra, whereas the traditionally used assumption of Δ(*T*, *φ*)≡*ɛ*_peak_(*T*, *φ*) is invalid. The self-energy (Σ=Σ′+*i*Σ′′) we use has a minimal representation with two different scattering rates, Γ_0_ and Γ_1_ (refs 32, 33),


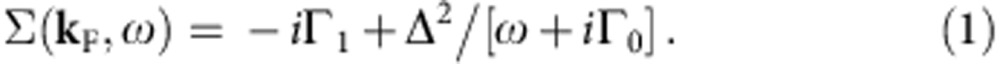


Here Γ_1_ is a single-particle scattering rate and it causes the broadening of a peak width. On the other hand, Γ_0_ fills the spectral weight around *E*_F_, and should be viewed as the inverse pair lifetime (or pair-breaking rate). For clarity, we label the former Γ_single_ and the latter Γ_pair_ in the rest of this paper. We emphasize that the intensity at *E*_F_ in a gapped spectrum becomes non-zero only when Γ_pair_≠0 as simulated in Supplementary Fig. 8a. Our spectra measured at the low temperatures (*T*≪*T*_c_) have a negligible intensity at *E*_F_, which ensures that our data are almost free from impurity-causing pair-breaking effect. At elevated temperatures, we observe a remarkable gap filling (see Fig. 1d and Supplementary Figs 2 and 5). Significantly, it actually begins from deep below *T*_c_, which is not expected in a conventional BCS superconductor. Since the data were measured at the extremely high energy resolution (Δ*ɛ*=1.4 meV), we can rule out the possibility, assumed before with setting Γ_pair_≡0 (refs 6, 15), that the filling is caused by a spectral broadening due to the experimental energy resolution. The intensity at *E*_F_ should instead be a signature of intrinsic pair breaking; hence, it must be taken into account for the gap estimation. In passing, we note that the Γ_single_ and Γ_pair_ both equally increase the intensity around *E*_F_ of the momentum-integrated spectrum previously studied by ARPES^9^,^18^,^19^ and STM^20^,^34^ (see Supplementary Fig. 9), which is therefore incapable of disentangling these two different scattering rates.

Following this consideration, we set Γ_single_ (or Γ_1_) and Γ_pair_ (or Γ_0_) to be independent free parameters in equation (1). First, we performed a spectral fitting to our ARPES data, assuming Δ(*T*)≡*ɛ*_peak_(*T*), which is a traditional way of gap estimation (Supplementary Figs 11a and 12a). The obtained parameter of Γ_single_(*T*) (middle panel of Supplementary Figs 11a and 12a) is strongly deviated from a monotonic decrease on cooling, having an unrealistic upturn around the temperature at which the *ɛ*_peak_ becomes zero. As already discussed above, this anomaly is expected when the spectrum with a single peak (*ɛ*_peak_=0) has an energy gap (Δ≠0); thus, the spectral width overestimates the scattering rate.

**Figure 5:**
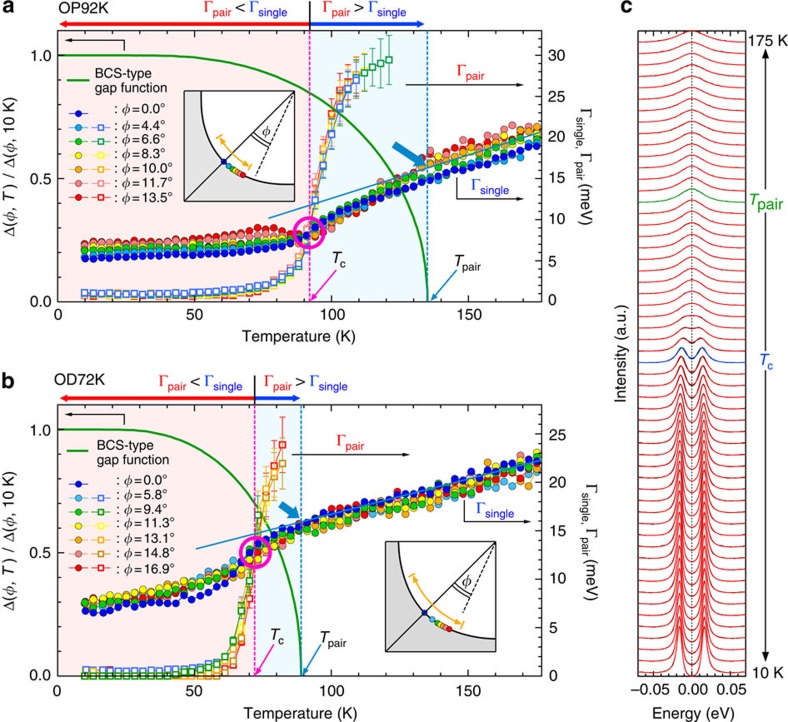
Extracted parameters of the minimal model spectral function required to reproduce the ARPES spectra. (**a**) The BCS-type gap function used for the fitting to the OP92K data (a green curve), and the obtained single-particle scattering rate (Γ_single_, or Γ_1_) and the pair-breaking rate (Γ_pair_, or Γ_0_) in equation (1). (**b**) The fitting results same as in **a**, but for the OD72K data. The values of Γ_pair_ at high temperatures are not plotted, since the spectral shape is insensitive to the Γ_pair_ when Δ is small or zero, and thus it is impossible to determine the value. A small hump seen in the Γ_pair_ around 75 K for *φ*=11.7° and 13.5° in **a** comes from a slight difficulty of fitting to the spectra with a peak-dip-hump shape due to the mode coupling, which appears below *T*_c_ and gets pronounced with approaching the antinode. Magenta circle marks the crossing point of Γ_single_(*T*) and Γ_pair_(*T*). Large blue arrow indicates the temperature, at which the Γ_single_(*T*) deviates from a *T*-linear behaviour on cooling. (**c**) ARPES spectra (black curves) and fitting results (red curves) providing the parameters for *φ*=13.5° in **a**. Error bars in **a** and **b** represent standard deviations in fitting the model spectral function to the ARPES spectra.

We find that this circumstance is corrected by applying a BCS-type gap function with an onset at 135 and 89 K for OP92K and OD72K (green curves in the bottom panels of Fig. 2a,b), respectively, regardless of the Fermi angle *φ*. In the Supplementary Figs 14 and 15, we fit equation (1) with such a gap function Δ to our ARPES data near the node measured over a wide temperature range. As an example, the result at *φ*=13.5° for OP92K is shown in Fig. 5c. The fitting curves (red curves) almost perfectly reproduce the data (black curves). The obtained Γ_single_(*T*) (Fig. 5a,b) in the gapped region agree with Γ_single_(*T*) at the node, which can be determined simply from the spectral width. Similarly, the Γ_pair_(*T*) curves are also almost identical for all the *φ*. The consistency in our results pointing to the isotropic scattering mechanism validates our model spectral function characterized by the BCS-type Δ(*T*, *φ*). The famous ‘hot spots', at which the scattering rate is abruptly enhanced, should be situated at much higher *φ* (Supplementary Fig. 13). The applied onset temperatures are almost the same as those of Nernst and diamagnetic effects^25^,^26^, which are viewed as signatures of phase-incoherent superconductivity. The comparable temperatures are also obtained by the specific heat measurements^35^ and the other spectroscopic techniques^36^,^37^. Therefore, we assign ∼135 and ∼89 K to be the onset temperature of pair formation (*T*_pair_) of OP92K and OD72K, respectively. This is further supported by the signature of pairing seen in the behaviour of Γ_single_(*T*) (Fig. 5a,b; Supplementary Fig. 16); the decrease of its value on cooling is accelerated across *T*_pair_, showing a deviation from the linear behaviour^38^. The different experimental techniques could have different sensitivities to the superconducting fluctuation above *T*_c_, and actually the terahertz spectroscopy estimates a slightly lower temperature scale (10–15 K above *T*_c_)^39^,^40^. Nonetheless, we stress that the view that the point nodal pairing survives above *T*_c_ is compatible in these observations. The doping variation of *T*_pair_/*T*_c_ (1.47 and 1.24 for OP92K and OD72K, respectively) obtained in our studies is consistent with the phase-fluctuating superconductivity, which merges to the superconducting dome with heavily overdoping^41^. The competing ordered phase is, in contrast, claimed to terminate at zero temperature inside the superconducting dome^42^, thus, disagrees with the present gapped states observed above *T*_c_ even in the overdoped sample.

### A simple formula determining the magnitude of *T*_c_ in cuprates

The relationship between Γ_pair_ and Γ_single_ should provide rich information relevant for the pairing mechanism. Intriguingly, the superconductivity occurs when the magnitude of Γ_pair_ is reduced smaller than that of Γ_single_; the *T*_c_ is coincident with the temperature at which Γ_single_(*T*) and Γ_pair_(*T*) crosses (magenta circles in Fig. 5a,b), which provides a simple formula of Γ_single_(*T*_c_)=Γ_pair_(*T*_c_). The magnitude of *T*_pair_ is reported to be comparable (120∼150 K) among different cuprate families with significantly different *T*_c_s^36^. The Γ_single_(*T*) also seems to be less sensitive to the different compounds^43^,^44^. Therefore, the pair-breaking effect, which controls the fulfillment of Γ_pair_<Γ_single_, is predicted to be a critical factor determining the *T*_c_ value of cuprates. Notably, a remarkable difference in filling behaviours of the spectral gap is indeed observed between Bi2212 and Bi_2_Sr_2_CuO_6+*δ*_ with about three times different *T*_c_s^9^,^36^.

## Discussion

**Figure 6:**
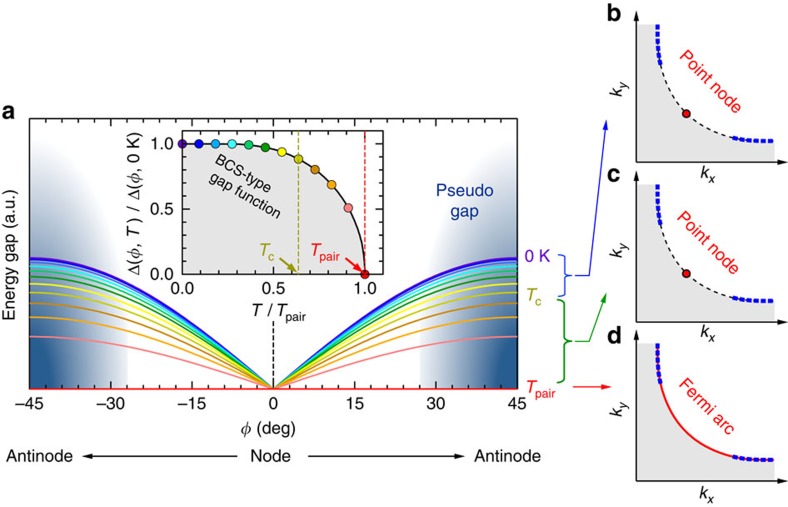
Schematic pairing gap evolution based on our ARPES results. (**a**) Temperature variation of the point nodal *d*-wave pairing gap. The energy gap has a BCS-type function (inset curve) with the same onset at *T*_pair_ regardless of directions (*φ*) along the Fermi surface. Temperatures for each curve are indicated in the inset with coloured circles. The gapped Fermi surface with a point node below *T*_c_ (**b**) persists beyond *T*_c_ (**c**) up to the temperature of pair formation (*T*_pair_). (**d**) Emergence of the gapless Fermi arc centered at the node due to the pseudogap evolution around the antinode^9^,^27^; while the antinodal region is not observable at the low photon energies like 7 eV, the studies with higher energy photons have demonstrated that the competing pseudogap state emerges at |*φ*|>25° in the optimal doping^9^.

We summarize our conclusion in Fig. 6 by drawing a schematic temperature evolution of the pairing gap. As demonstrated elsewhere^9^, the pseudogap competing with the pairing disappears far off the node (blue area). In this article, we have investigated the nodal region of optimally and overdosed samples. We revealed that the point node state persists up to far above *T*_c_, against the previous expectation^15^,^16^,^17^,^20^,^21^,^24^, following the BCS-type gap function with the onset at *T*_pair_ (∼1.5*T*_c_ in the optimal doping). The ARPRS spectra are reproduced by the spectral function with a minimal model, which has a single energy gap all the way up to the gap closing temperature. It is consistent with the expected ‘pure' pairing state with no contamination by the pseudogap around the node at least in the optimally and overdosed regions. While the gap evolution with an onset at *T*_pair_ might be reminiscent of a phase-transition phenomenon, it is against a gradual temperature variation of the specific heat^35^. The crossover-like behaviours in cuprates should come from the significant pair-breaking effect, which is markedly enhanced above T_c_. To fully understand the present results, insight of the spacially inhomogeneous state^45^ would be essential. However, only that cannot explain our data, since the local density of states itself has the behaviour of gap filling at *E*_F_ with temperature^34^. The competing nature of pseudogap state evolving around the antinode^1^,^3^ is a plausible source for the unique scattering mechanism, which strongly suppress *T*_c_ from *T*_pair_. To evaluate this speculation, however, the more detailed theoretical inputs are required.

## Methods

### Samples

Optimally doped Bi_2_Sr_2_CaCu_2_O_8+*δ*_ (OP92 K) and overdoped (Bi,Pb)_2_Sr_2_CaCu_2_O_8+*δ*_ (OD72 K) single crystals with *T*_c_=92 and 72 K, respectively, were grown by the conventional floating-zone technique. A sharp superconducting transition width of ∼1 K (OP92K) and ∼3 K (OD72K) were confirmed (see Supplementary Fig. 1).

### ARPES experiments

ARPES data were accumulated using a laboratory-based system consisting of a Scienta R4000 electron analyser and a 6.994 eV laser. The overall energy resolution in the ARPES experiment was set to 1.4 meV for all the measurements. To accomplish the temperature scan of spectra at a high precision, we applied a technique of the local sample heating, which thermally isolates the sample holder with a heat switch from the lest of the system at elevated temperatures. It minimizes the degassing, allowing us to keep the chamber pressure better than 2 × 10^−11^ torr during the entire temperature sweeping; no sample aging was confirmed (Supplementary Fig. 4). This method also prevents the thermal expansion of sample manipulator, and it enables us to take data in fine temperature steps with automated measurement of temperature scan from precisely the same spot on the crystal surface, which was essential to achieve the aim of the present study.

## Additional information

**How to cite this article**: Kondo, T. *et al.* Point nodes persisting far beyond *T*_c_ in Bi2212. *Nat. Commun.* 6:7699 doi: 10.1038/ncomms8699 (2015).

## Supplementary Material

Supplementary Information Supplementary Figures 1-17, Supplementary Notes 1-9 and Supplementary References

## References

[b1] Kondo T., Khasanov R., Takeuchi T., Schmalian J. & Kaminski A. Competition between the pseudogap and superconductivity in the high-*T*_*c*_ copper oxides. Nature 457, 296–300 (2009). 19148096 10.1038/nature07644

[b2] Khasanov R. *et al.* Evidence for a competition between the superconducting state and the pseudogap state of (BiPb)_2_(SrLa)_2_CuO_6+*δ*_ from muon spin rotation experiments. Phys. Rev. Lett. 101, 227002 (2008). 19113513 10.1103/PhysRevLett.101.227002

[b3] Chang J. *et al.* Direct observation of competition between superconductivity and charge density wave order in YBa_2_Cu_3_O_6.67_. Nat. Phys. 8, 871–876 (2012).

[b4] Hashimoto M. *et al.* Particle-hole symmetry breaking in the pseudogap state of Bi2201. Nat. Phys. 6, 414–418 (2010).

[b5] Wise W. D. *et al.* Charge-density-wave origin of cuprate checkerboard visualized by scanning tunnelling microscopy. Nat. Phys. 4, 696–699 (2008).

[b6] Vishik I. M. *et al.* Phase competition in trisected superconducting dome. Proc. Natl Acad. Sci. USA 109, 18332–18337 (2012). 23093670 10.1073/pnas.1209471109 PMC3494935

[b7] Parker C. V. *et al.* Fluctuating stripes at the onset of the pseudogap in the high-*T*_*c*_ superconductor Bi_2_Sr_2_CaCu_2_O_8+*δ*_. Nature 468, 677–680 (2010). 21124453 10.1038/nature09597

[b8] He R.-H. *et al.* From a single-band metal to a high-temperature superconductor via two thermal phase transitions. Science 331, 1579–1583 (2011). 21436447 10.1126/science.1198415

[b9] Kondo T. *et al.* Formation of gapless fermi arcs and fingerprints of order in the pseudogap state of cuprate superconductors. Phys. Rev. Lett. 111, 157003 (2013). 24160620 10.1103/PhysRevLett.111.157003

[b10] Comin R. *et al.* Charge order driven by Fermi-arc instability in Bi_2_Sr_2−*x*_La_*x*_CuO_6+*δ*_. Science 343, 390–392 (2014). 24356115 10.1126/science.1242996

[b11] McElroy K. *et al.* Coincidence of checkerboard charge order and antinodal state decoherence in strongly underdoped superconducting Bi_2_Sr_2_CaCu_2_O_8+*δ*_. Phys. Rev. Lett. 94, 197005 (2005). 16090202 10.1103/PhysRevLett.94.197005

[b12] da Silva Neto E. H. *et al.* Ubiquitous interplay between charge ordering and high-temperature superconductivity in cuprates. Science 343, 393–396 (2014). 24356110 10.1126/science.1243479

[b13] RazzoliE. *et al.* Evolution from a nodeless gap to 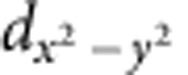 -wave in underdoped La_2−*x*_Sr_*x*_CuO_4_. Phys. Rev. Lett. 110, 047004 (2013).2516619610.1103/PhysRevLett.110.047004

[b14] Peng Y. *et al.* Disappearance of nodal gap across the insulator–superconductor transition in a copper-oxide superconductor. Nat. Commun. 4, 2459 (2013). 24051514 10.1038/ncomms3459

[b15] Lee W. S. *et al.* Abrupt onset of a second energy gap at the superconducting transition of underdoped Bi2212. Nature 450, 81–84 (2007). 17972881 10.1038/nature06219

[b16] Kanigel A. *et al.* Evolution of the pseudogap from Fermi arcs to the nodal liquid. Nat. Phys. 2, 447–451 (2006).

[b17] Nakayama K. *et al.* Evolution of a Pairing-Induced Pseudogap from the Superconducting Gap of (Bi,Pb)_2_Sr_2_CuO_6_. Phys. Rev. Lett. 102, 227006 (2009). 19658895 10.1103/PhysRevLett.102.227006

[b18] Reber T. J. *et al.* The origin and non-quasiparticle nature of Fermi arcs in Bi_2_Sr_2_CaCu_2_O_8+*δ*_. Nat. Phys. 8, 606–610 (2012).

[b19] Reber T. J. *et al.* Prepairing and the ‘filling' gap in the cuprates from the tomographic density of states. Phys. Rev. B 87, 060506 (2013).

[b20] Pushp A. *et al.* Extending universal nodal excitations optimizes superconductivity in Bi_2_Sr_2_CaCu_2_O_8+*δ*_. Science 324, 1689–1693 (2009). 19498107 10.1126/science.1174338

[b21] Lee J. *et al.* Spectroscopic fingerprint of phase-incoherent superconductivity in the underdoped Bi_2_Sr_2_CaCu_2_O_8+*δ*_. Science 325, 1099–1103 (2009). 19713522 10.1126/science.1176369

[b22] Boyer M. *et al.* Imaging the two gaps of the high-temperature superconductor Bi_2_Sr_2_CuO_6+x_. Nat. Phys. 3, 802–806 (2007).

[b23] Norman M. R. *et al.* Destruction of the Fermi surface in underdoped high-*T*_*c*_ superconductors. Nature 392, 157–160 (1998).

[b24] Kanigel A. *et al.* Protected nodes and the collapse of fermi arcs in high-*T*_*c*_ cuprate superconductors. Phys. Rev. Lett. 99, 157001 (2007). 17995204 10.1103/PhysRevLett.99.157001

[b25] Wang Y., Li L. & Ong N. Nernst effect in high-*T*_*c*_ superconductors. Phys. Rev. B 73, 024510 (2006).

[b26] Wang Y. *et al.* Field-enhanced diamagnetism in the pseudogap state of the cuprate Bi_2_Sr_2_CaCu_2_O_8+*δ*_ superconductor in an intense magnetic field. Phys. Rev. Lett. 95, 247002 (2005). 16384409 10.1103/PhysRevLett.95.247002

[b27] Kohsaka Y. *et al.* How Cooper pairs vanish approaching the Mott insulator in Bi_2_Sr_2_CaCu_2_O_8+*δ*_. Nature 454, 1072–1078 (2008). 18756248 10.1038/nature07243

[b28] Hanaguri T. *et al.* Quasiparticle interference and superconducting gap in Ca_2−*x*_Na_*x*_CuO_2_Cl_2_. Nat. Phys. 3, 865–871 (2007).

[b29] Kiss T. *et al.* A versatile system for ultrahigh resolution, low temperature, and polarization dependent Laser-angle-resolved photoemission spectroscopy. Rev. Sci. Instrum. 79, 023106–023106 (2008). 18315282 10.1063/1.2839010

[b30] Kordyuk A., Borisenko S., Knupfer M. & Fink J. Measuring the gap in angle-resolved photoemission experiments on cuprates. Phys. Rev. B 67, 064504 (2003).

[b31] Varma C. M. & Zhu L. Topological transition in the fermi surface of cuprate superconductors in the pseudogap regime. Phys. Rev. Lett. 98, 177004 (2007).

[b32] Chubukov A. V., Norman M. R., Millis A. J. & Abrahams E. Gapless pairing and the Fermi arc in the cuprates. Phys. Rev. B 76, 180501 (2007).

[b33] Norman M. R., Randeria M., Ding H. & Campuzano J. C. Phenomenology of the low-energy spectral function in high-*T*_*c*_ superconductors. Phys. Rev. B 57, R11093 (1998).

[b34] Pasupathy A. N. *et al.* Electronic origin of the inhomogeneous pairing interaction in the high-*T*_*c*_ superconductor Bi_2_Sr_2_CaCu_2_O_8+*δ*_. Science 320, 196–201 (2008). 18403704 10.1126/science.1154700

[b35] Tallon J. L., Storey J. G. & Loram J. W. Fluctuations and critical temperature reduction in cuprate superconductors. Phys. Rev. B 83, 092502 (2011).

[b36] Kondo T. *et al.* Disentangling Cooper-pair formation above the transition temperature from the pseudogap state in the cuprates. Nat. Phys. 7, 21–25 (2011).

[b37] Gomes K. K. *et al.* Visualizing pair formation on the atomic scale in the high-*T*_*c*_ superconductor Bi_2_Sr_2_CaCu_2_O_8+*δ*_. Nature 447, 569–572 (2007). 17538615 10.1038/nature05881

[b38] Barišic′ N. *et al.* Universal sheet resistance and revised phase diagram of the cuprate high-temperature superconductors. Proc. Natl Acad. Sci. USA 27, 8424 (2013). 10.1073/pnas.1301989110 PMC3725090 23836669

[b39] Orenstein J., Corson J., Oh S. & Eckstein J. Superconducting fluctuations in Bi_2_Sr_2_Ca_1−*x*_Dy_*x*_Cu_2_O_8+*δ*_ as seen by terahertz spectroscopy. Annalen der Physik 15, 596–605 (2006).

[b40] Bilbro L. *et al.* Temporal correlations of superconductivity above the transition temperature in La_2−*x*_Sr_*x*_CuO_4_ probed by terahertz spectroscopy. Nat. Phys. 7, 298–302 (2011).

[b41] Emery V. & Kivelson S. Importance of phase fluctuations in superconductors with small superfluid density. Nature 374, 434–437 (1995).

[b42] Shekhter A. *et al.* Bounding the pseudogap with a line of phase transitions in YBa_2_Cu_3_O_*δ*_. Nature 498, 75–77 (2013). 23739425 10.1038/nature12165

[b43] Kondo T. *et al.* Anomalous doping variation of the nodal low-energy feature of superconducting (Bi,Pb)_2_(Sr,La)_2_CuO_6+*δ*_ crystals revealed by laser-based angle-resolved photoemission spectroscopy. Phys. Rev. Lett. 110, 217006 (2013). 23745917 10.1103/PhysRevLett.110.217006

[b44] Zhou X. J. *et al.* High-temperature superconductors: universal nodal Fermi velocity. Nature 423, 398–398 (2003). 12761537 10.1038/423398a

[b45] Pan S. H. *et al.* Microscopic electronic inhomogeneity in the high-*T*_*c*_ superconductor Bi_2_Sr_2_CaCu_2_O_8+*x*_. Nature 413, 282–285 (2001). 11565024 10.1038/35095012

