# Supplementary material for: Point nodes persisting far beyond Tc in Bi2212
Source: Nat Commun. 2015 Jul 9;6:7699. doi: 10.1038/ncomms8699 (PMC4510699; doi:10.1038/ncomms8699)
Supplement: Supplementary Information — Supplementary Figures 1-17, Supplementary Notes 1-9 and Supplementary References [file ncomms8699-s1.pdf]

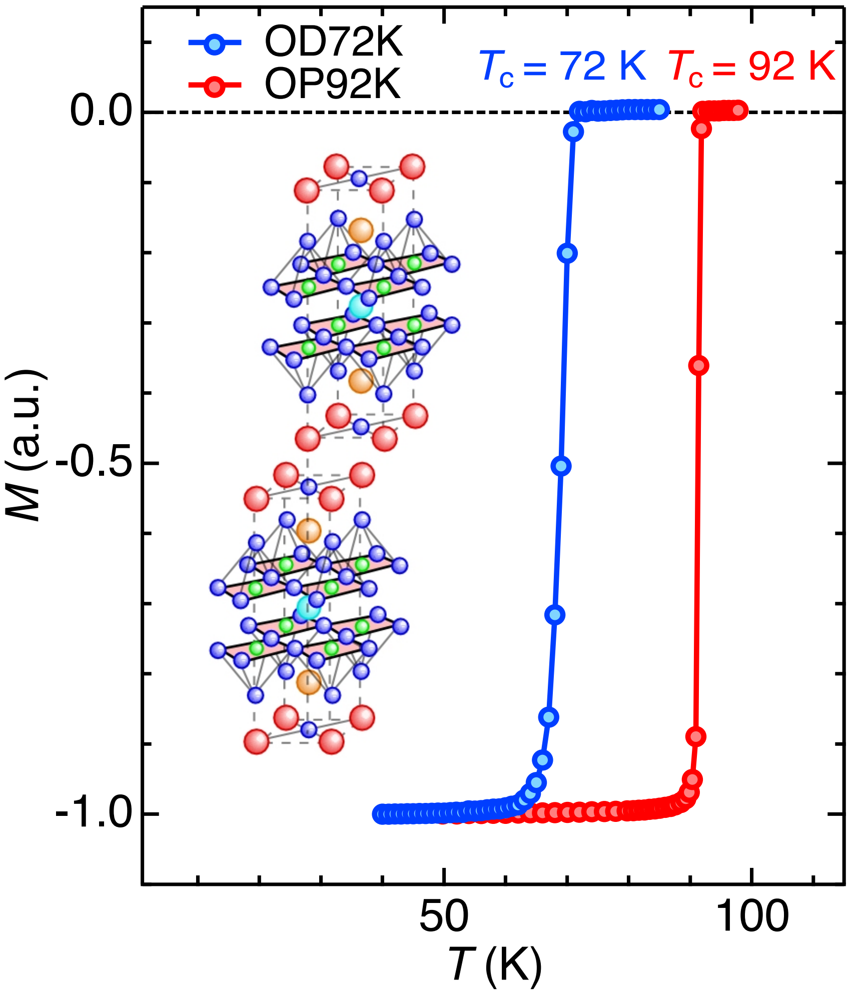

Supplementary Figure 1: Magnetic susceptibility for single crystals of optimally doped $Bi_2Sr_2CaCu_2O_{8+\delta}$ (OP92K) and overdoped $(Bi,Pb)_2Sr_2CaCu_2O_{8+\delta}$ (OD72K) with an onset T_c of 92K and 72K, respectively, used for the ARPES measurements. The crystal structure of Bi2212 is drawn in the inset.

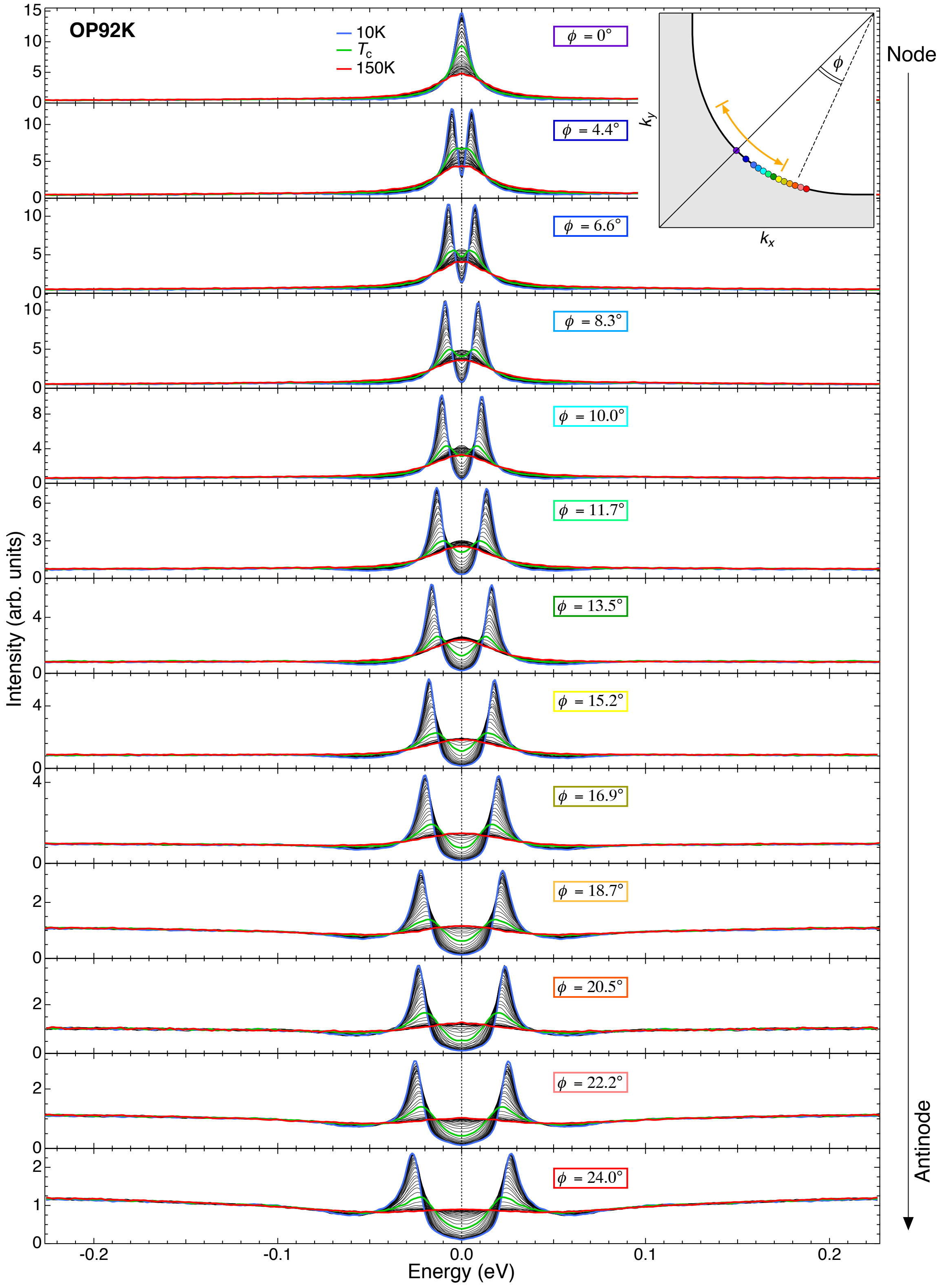

Supplementary Figure 2: Temperature evolution of ARPES spectra (symmetrized EDCs) at various $\mathbf{k}_{\mathrm{F}}$s for OP92K (color circles in the inset). The corresponding ϕ angles (defined in the inset) are described in each panel. The orange arrow in the inset indicates the momentum region where the gapless Fermi arc was previously claimed to emerge at T_{c}.

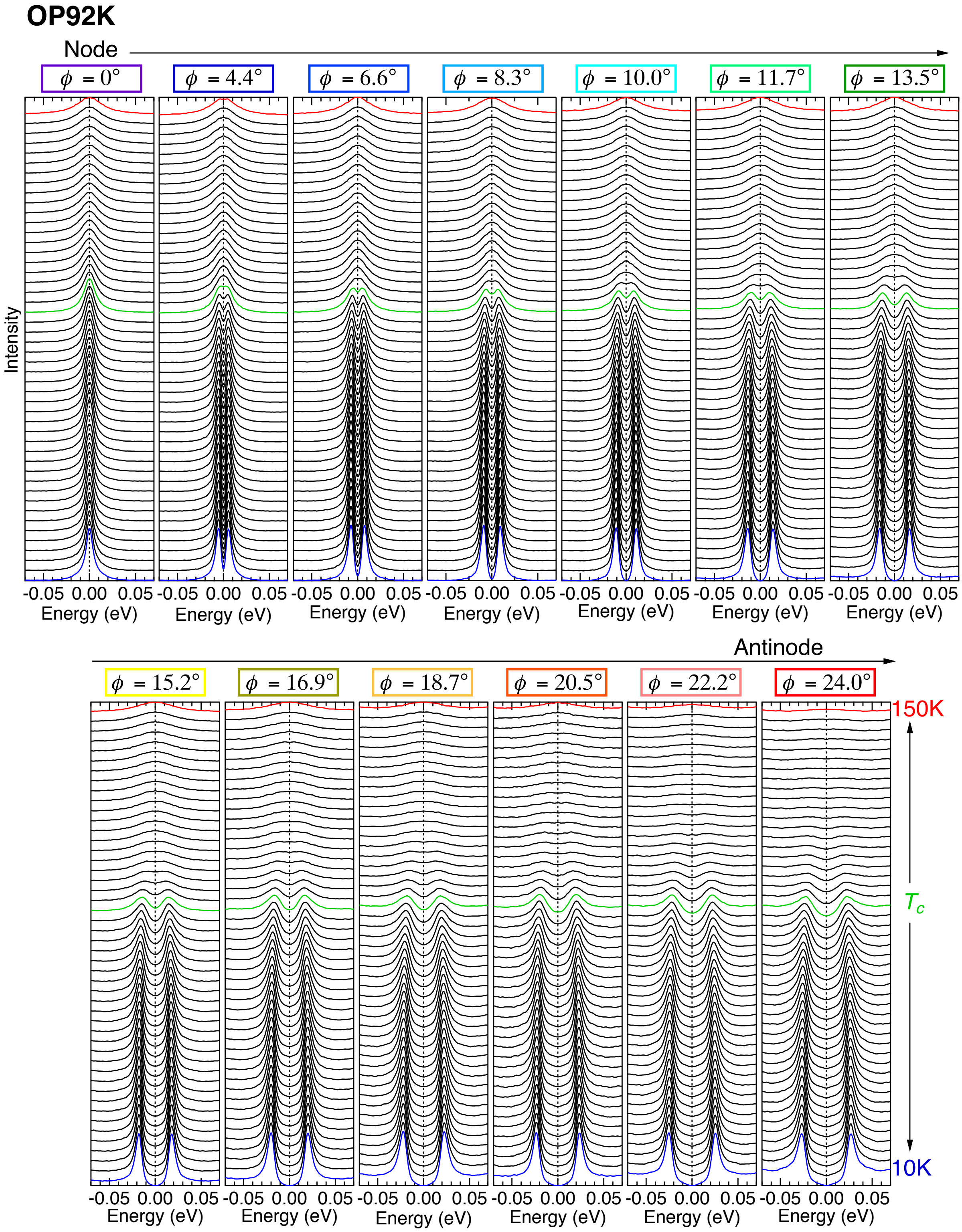

Supplementary Figure 3: Temperature evolution of ARPES spectra (symmetrized EDCs) at various $\mathbf{k}_{\mathrm{F}}$s for OP92K: the same data as in Supplementary Figure **2**, but plotted with an offset. The top left panel plots the data at the node ($\phi = 0°$). Toward the bottom right, the measured $\mathbf{k}_{\mathrm{F}}$ approaches the antinode. The blue, green, and red curves in each panel are the spectra at 10K, T_{c} (=92K), and 150K, respectively.

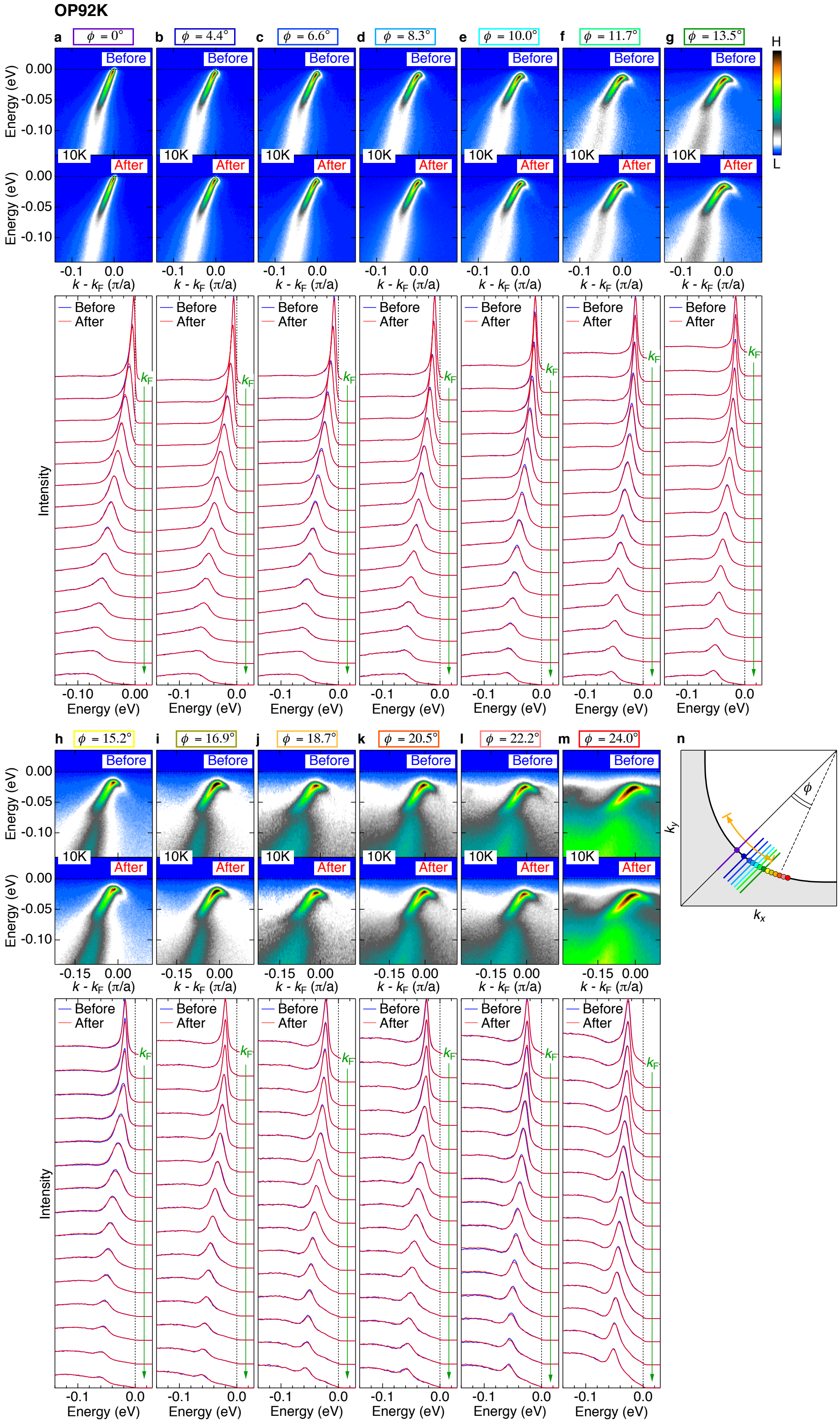

Supplementary Figure 4: Aging check of the sample surface after temperature scan. The upper left panels in **a** show the data along the node ($\phi = 0°$). Toward the bottom right in **m**, the measured momentum approaches the antinode. **n**, Fermi surface with the measured momentum cuts and $\mathbf{k}_F$ points.

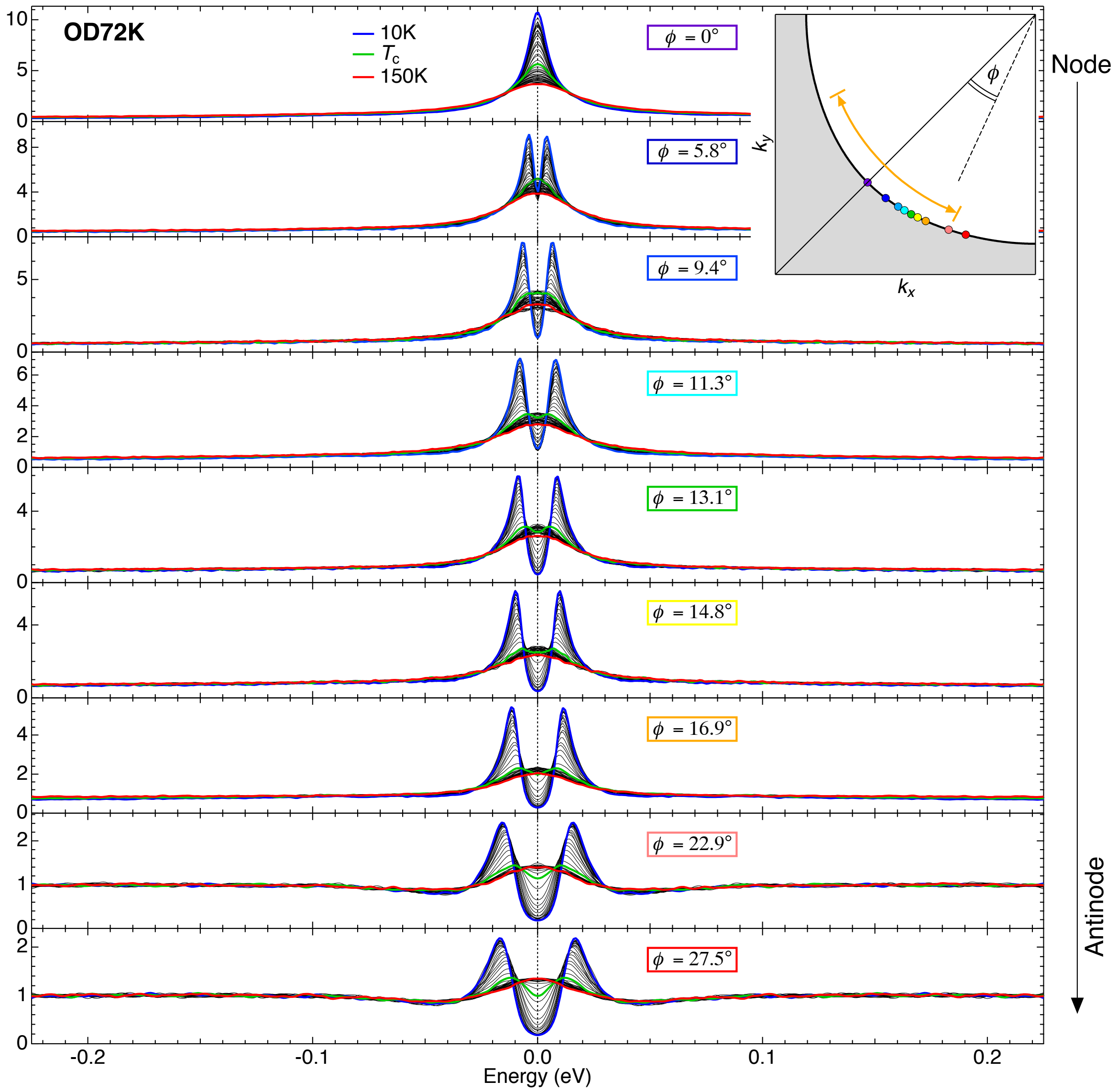

Supplementary Figure 5: Temperature evolution of ARPES spectra (symmetrized EDCs) at various $\mathbf{k}_\mathrm{F}$s for OD72K (color circles in the inset). The corresponding ϕ angles (defined in the inset) are described in each panel. The orange arrow in the inset indicates the momentum region where the gapless Fermi arc was previously claimed to emerge at T_c.

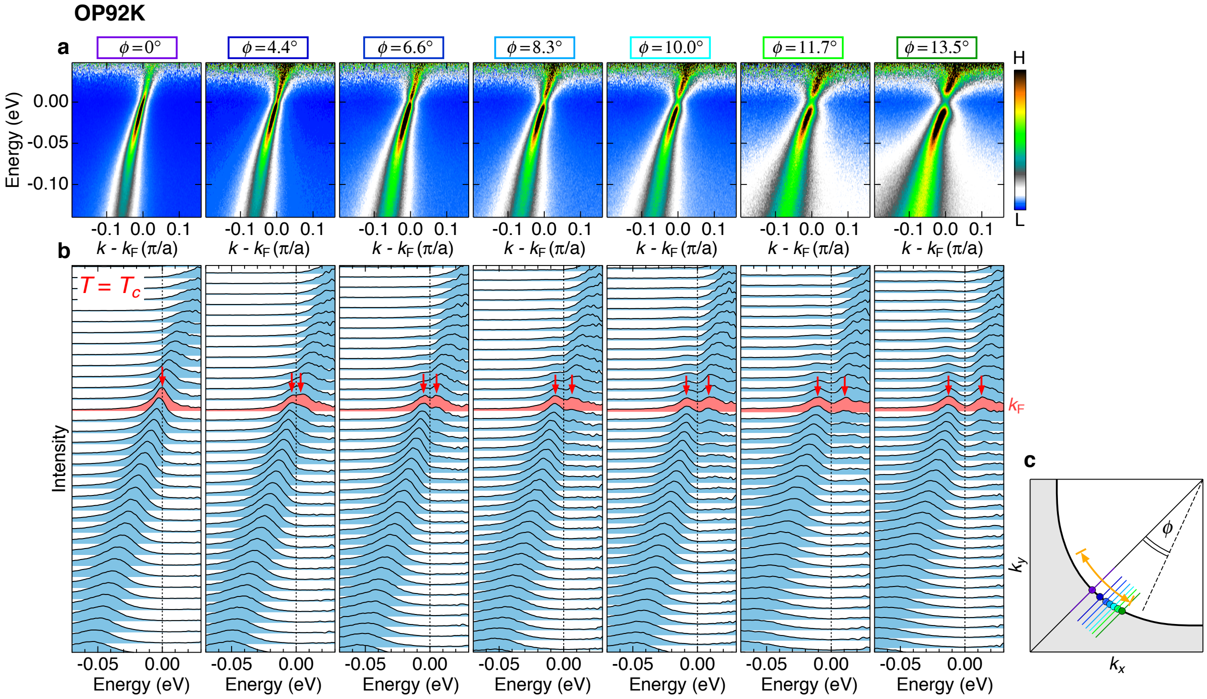

Supplementary Figure 6: Evidence for the absence of the Fermi arc at T_c. **a**, Fermi-function divided band dispersions at T_c along several momentum cuts (color lines in **c**). Same data as Fig.3a in the main paper. **b**, The corresponding EDCs. The spectra at $\mathbf{k}_F$s (color circles in **c**) are painted with red. Peak positions are indicated with red arrows. **c**, The Fermi surface. The orange arrow indicates the momentum region where the gapless Fermi arc was previously claimed to emerge at T_c.

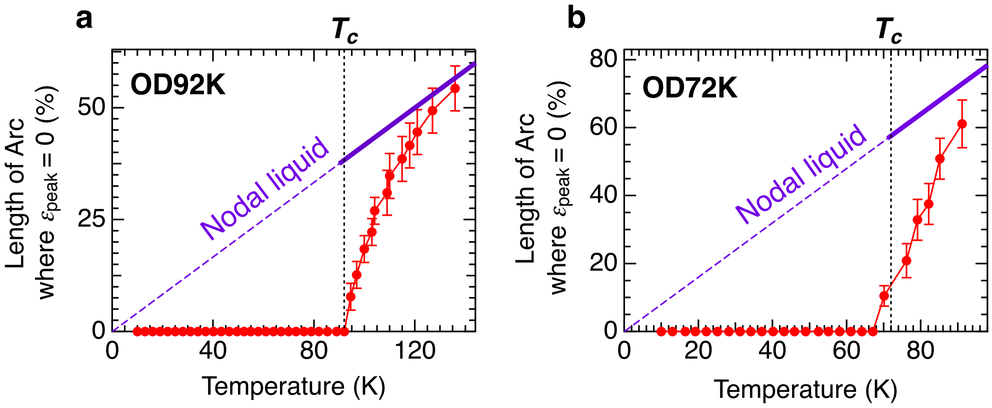

Supplementary Figure 7: The "artificial" Fermi arc. The length of arc where the symmetrized EDCs have a single-peak ($\varepsilon_{\rm peak}$=0) for OP92K (**a**) and OD72K (**b**). The nodal liquid behavior, which has a T-linear fashion and is extrapolated to zero at $T = 0$, is superimposed in each panel.

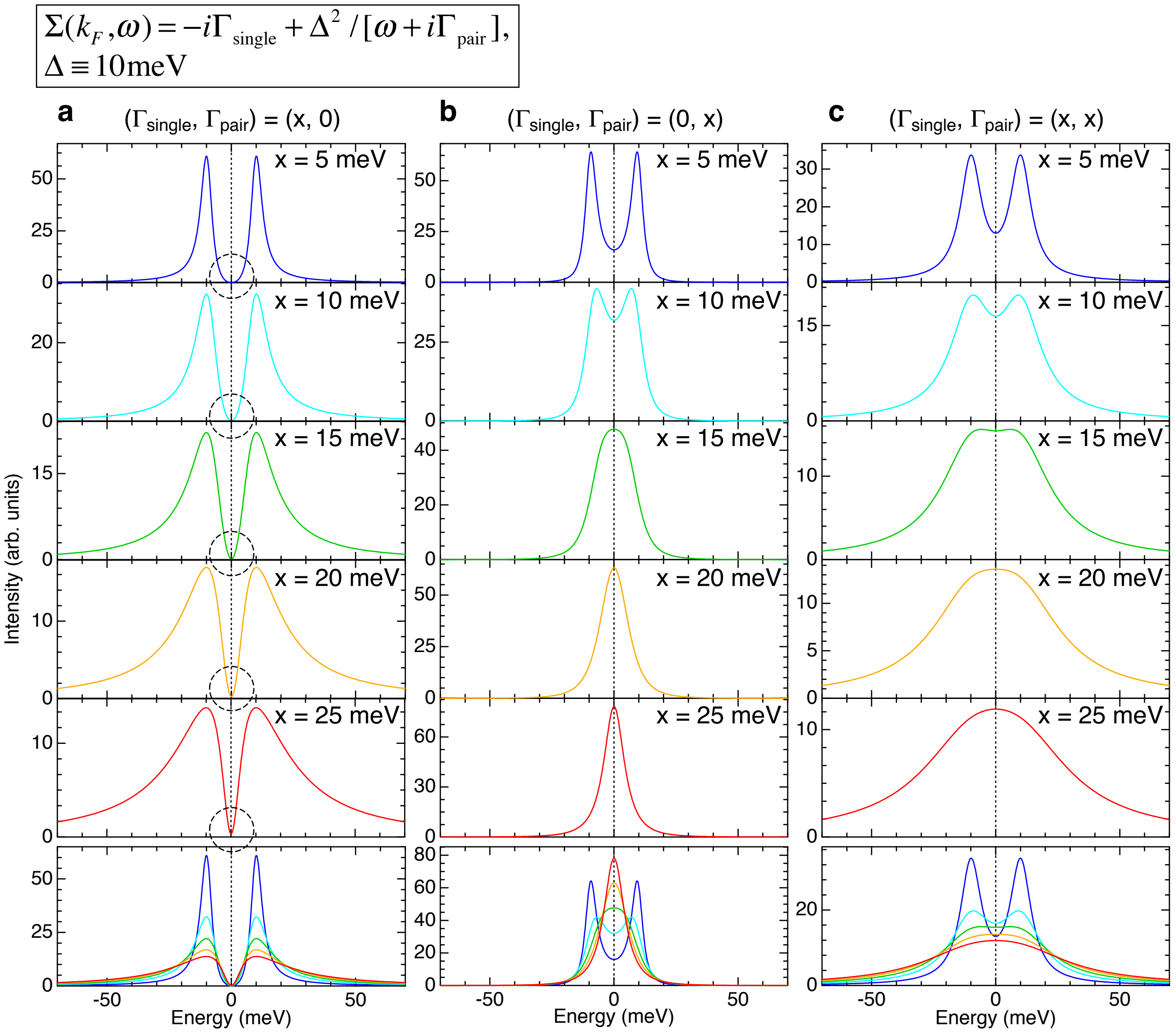

Supplementary Figure 8: Simulation of spectral function in Eq.(S1) for examining the effect of two different scattering rates (Γ_{single} and Γ_{pair}) on the spectral shape. In all the curves presented, the gap magnitude is set to 10meV ($\Delta \equiv 10$ meV). **a**, The Γ_{single} dependence when $\Gamma_{\text{pair}} \equiv 0$meV. **b**, The Γ_{pair} dependence when $\Gamma_{\text{single}} \equiv 0$meV. **c**, The spectral shape for various values of Γ_{single} and Γ_{pair}, which are set to be equal. In each bottom panel of **a**, **b**, and **c**, all spectra in the upper panels are superimposed. While all the curves plotted are convoluted with a gaussian that has the width of the experimental energy resolution ($\Delta\varepsilon = 1.4$meV), the $\Delta\varepsilon$ value is so small that the difference in shape from original curves is negligible.

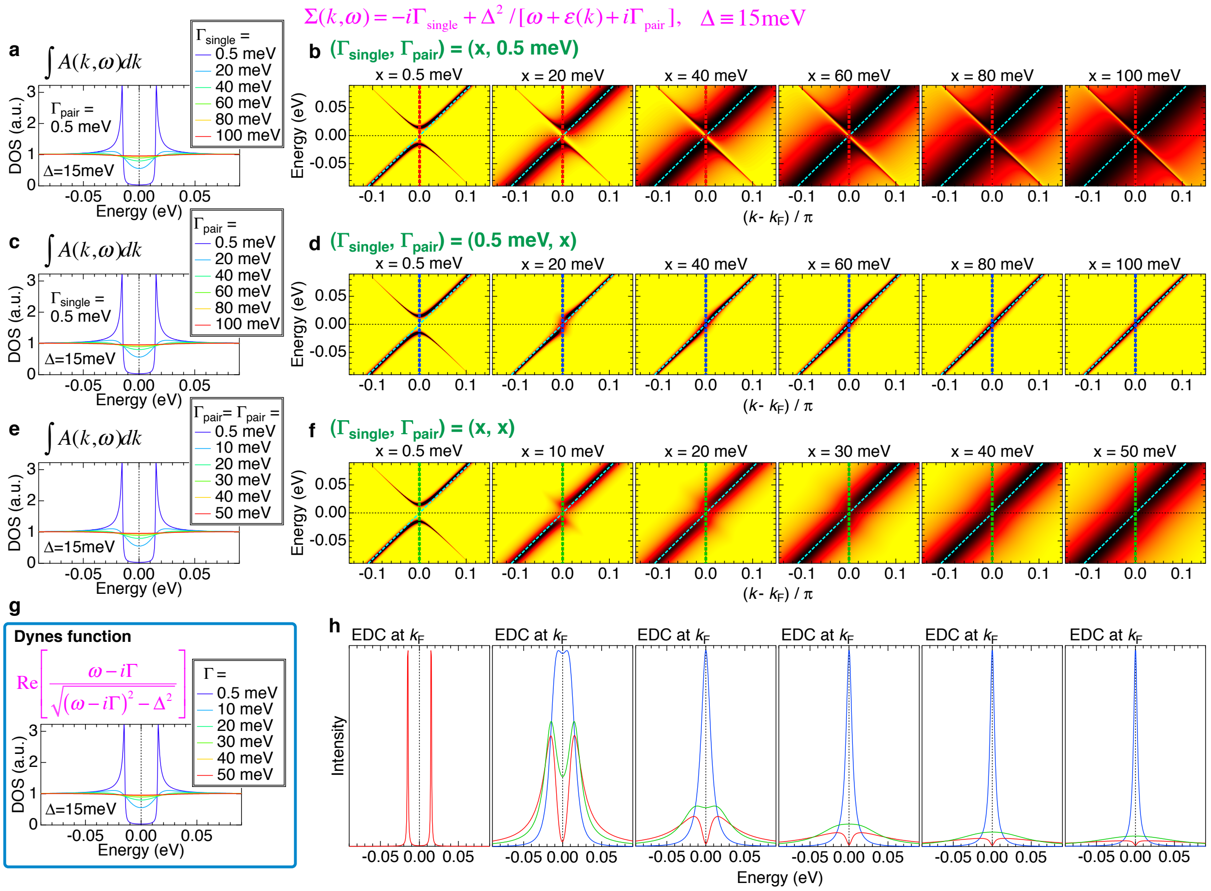

Supplementary Figure 9: Simulations for examining the effect of $\Gamma_{\rm single}$ and $\Gamma_{\rm pair}$ on the momentum-integrated spectra. The magnitude of energy gap Δ is fixed to 15 meV for all the cases. **a, c, e**, Momentum integrated spectra, $I_{\rm DOS}(\omega) = \int A(\mathbf{k},\omega)dk$, for the dispersion images in **b, d, f**, respectively. **b, d, f**, Dispersion images for one-particle spectral function, $A(\mathbf{k},\omega)$, in Eq.(S1) with a linear $\varepsilon(k)$ (light blue dashed lines). $\Gamma_{\rm single}$ and $\Gamma_{\rm pair}$ dependences are studied in (**a, b**) and (**c, d**), respectively. The case under the condition of $\Gamma_{\rm single} = \Gamma_{\rm pair}$ is simulated in (**e, f**). **g**, The spectra of Dynes function with several values of Γ. **h**, Spectra at $\mathbf{k}_{\rm F}$, $A(\mathbf{k}_{\rm F},\omega)$, extracted from images of **b, d, f**. Each color (red, blue, and green) in curves corresponds to that of thick dashed line added in the images of **b, d, f**.

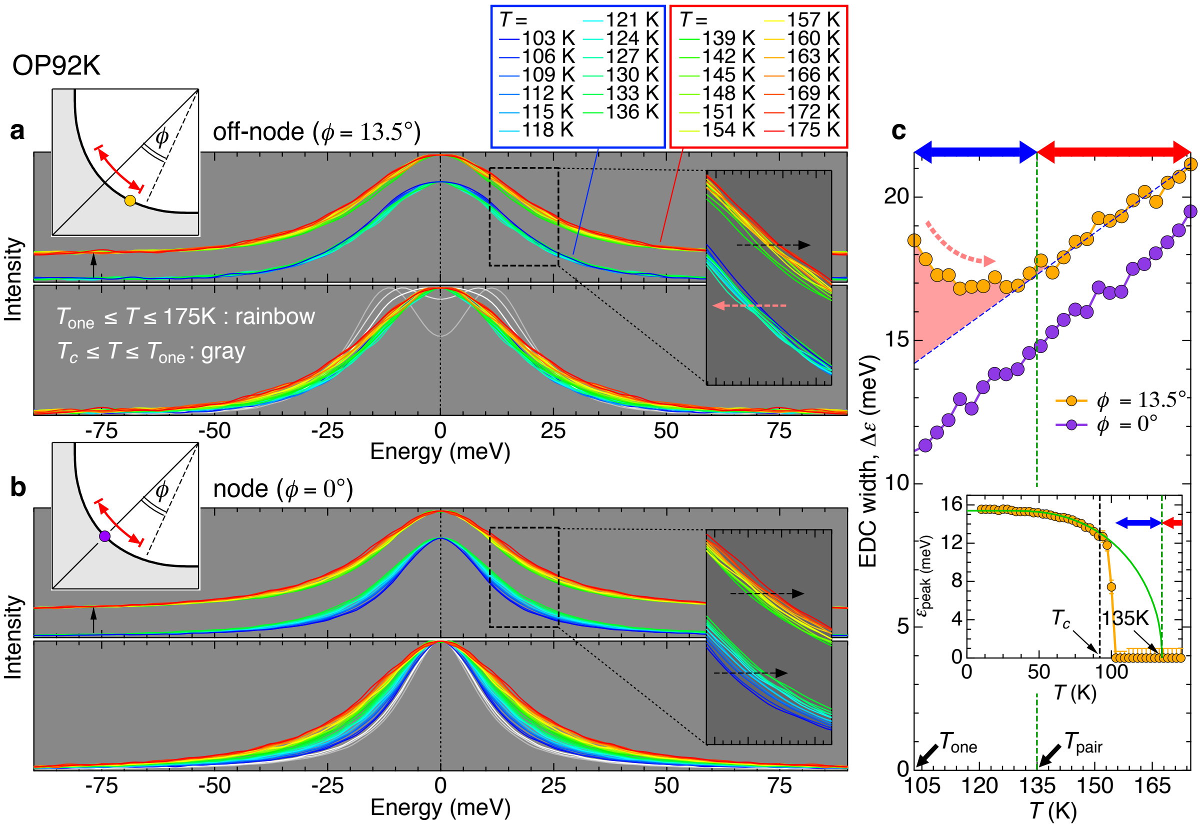

Supplementary Figure 10: Signature for the gap existence in the single-peak spectra off the node. Symmetrized EDCs of OP92K at $T > T_c$ measured off the node (bottom panel of **a**) and at the node (bottom panel of **b**). The $\mathbf{k}_F$ points are marked with circles in the inset of **a** ($\phi = 13.5°$) and **b** ($\phi = 0°$). Two peaks in the off-nodal spectra merge to one peak at 103K (T_{one}). **c**, The spectral width ($\Delta\varepsilon$) at $T > T_{one}$ are plotted as a function of temperature. The off-nodal $\Delta\varepsilon(T)$ decreases with temperature and reaches the minimum at $\sim$ 135K (T_{pair}) before it eventually increases at elevated temperatures. The anomalous behavior contrasts to the nodal $\Delta\varepsilon(T)$, which monotonically increases over the whole range of temperatures. In the upper panels of **a** and **b**, the spectra above T_{one} with a single-peak (rainbow colors) are extracted. In addition, the spectra at $T > T_{pair}$ and $T < T_{pair}$ are separated with an offset (black arrow). The insets show the magnified areas marked with the dashed squares. The black and magenta dashed arrows indicate the energy shifts of spectral tails with increasing temperature. The inset of **c** plots the energy positions of symmetrized EDCs (ε_{peak}) as a function of temperature. The green curve indicates the BCS-type gap function with the onset of 135K, which fits well the $\varepsilon_{peak}(T)$ at the low temperatures.

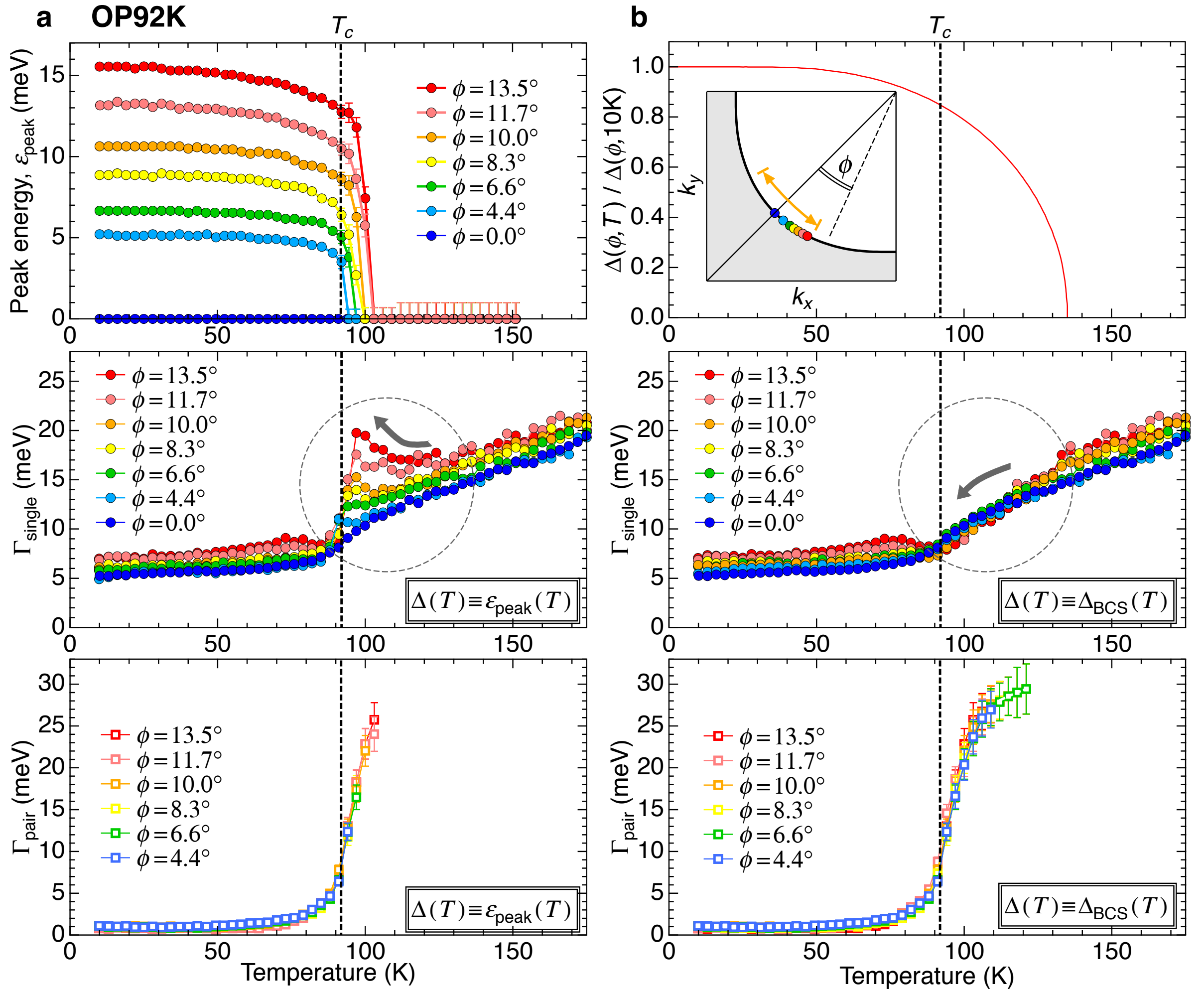

Supplementary Figure 11: Comparison of fitting results with Eq.(S1) for OP92K data between two different settings to the energy gap, Δ. **a**, The Δ is defined to be equal to the peak position of spectra [$\Delta(T) \equiv \varepsilon_{\rm peak}(T)$], shown in the top panel. **b**, The Δ is defined to have a BCS-type gap function with an onset at $T_{\rm pair}$=135K, shown in the top panel. The middle and bottom panels plot the obtained fitting parameters, $\Gamma_{\rm single}$ and $\Gamma_{\rm pair}$, at several $\mathbf{k}_{\rm F}$ points in the momentum region, where the gapless Fermi arc was previously claimed to emerge at $T_{\rm c}$ (see circles and an arrow in the inset of **b**). The values of $\Gamma_{\rm pair}$ at high temperatures are not plotted, since the spectral shape is not sensitive to the $\Gamma_{\rm pair}$ when Δ is small or zero, and thus it is impossible to precisely determine the value.

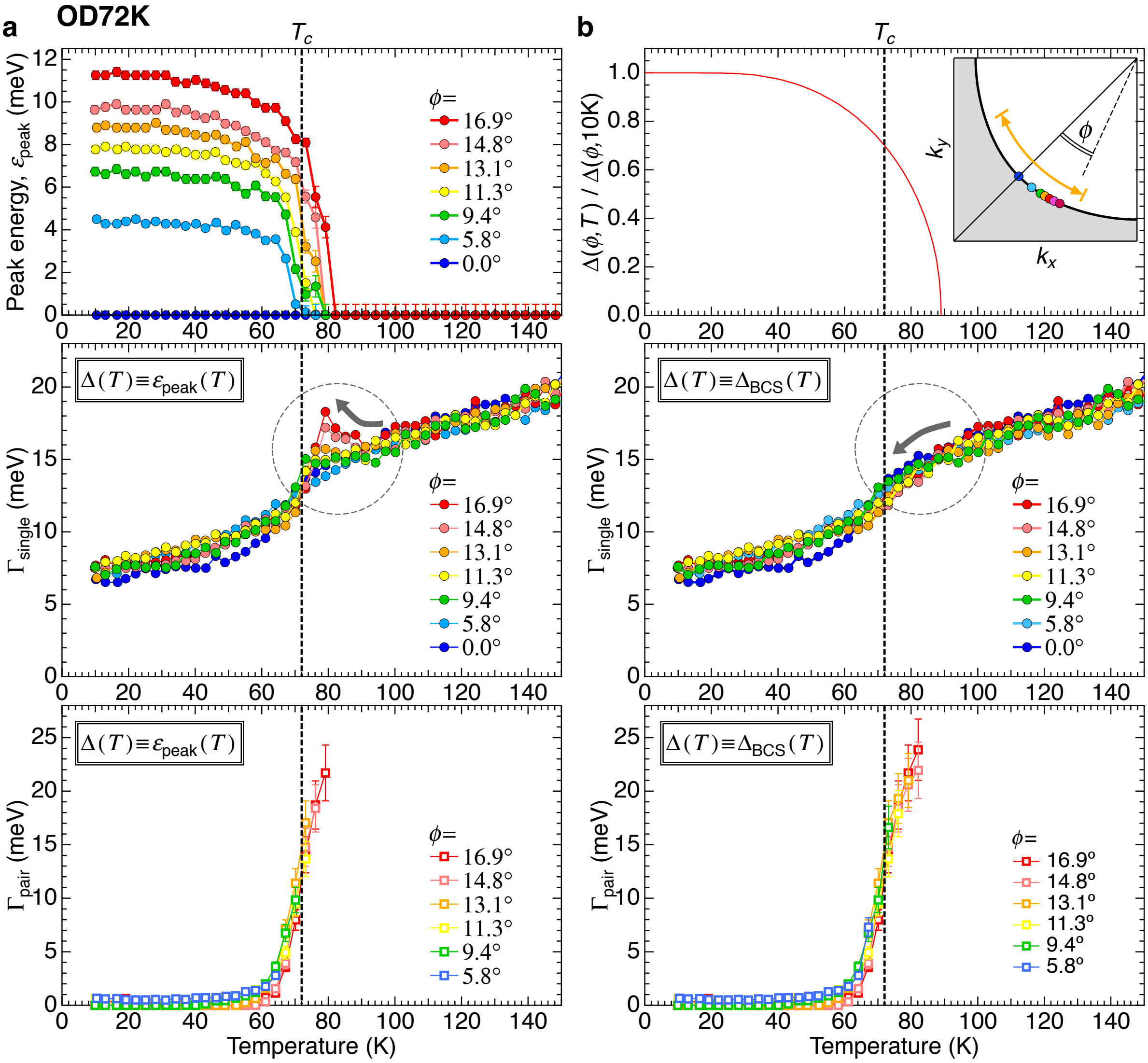

Supplementary Figure 12: Comparison of fitting results with Eq.(S1) for OD72K data between two different settings to the energy gap, Δ. **a**, The Δ is defined to be equal to the peak position of spectra $[\Delta(T) \equiv \varepsilon_{\text{peak}}(T)]$, shown in the top panel. **b**, The Δ is defined to have a BCS-type gap function with an onset at T_{pair}=89K, shown in the top panel. The middle and bottom panels plot the obtained fitting parameters, Γ_{single} and Γ_{pair}, at several $\mathbf{k}_{\text{F}}$ points in the momentum region, where the gapless Fermi arc was previously claimed to emerge at T_{c} (see circles and an arrow in the inset of **b**). The values of Γ_{pair} at high temperatures are not plotted, since the spectral shape is not sensitive to the Γ_{pair} when Δ is small or zero, and thus it is impossible to precisely determine the value.

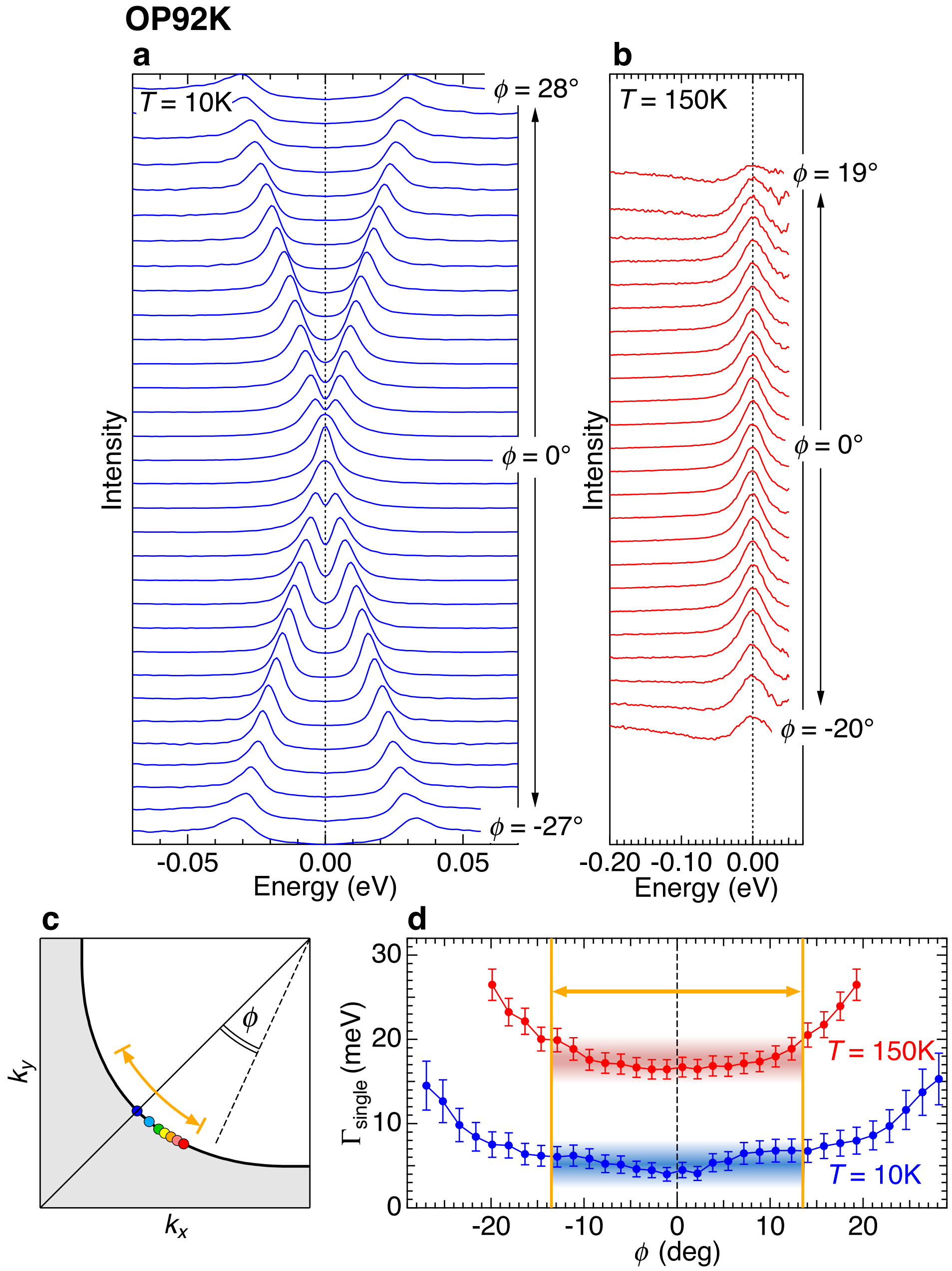

Supplementary Figure 13: Isotropic scattering mechanism around the node. **a**, **b**, ARPES spectra of OP92K measured at various $\mathbf{k}_\mathrm{F}$ points over a wide ϕ angle measured far below T_c (T=10K) (**a**) and above T_pair (T=150K) (**b**). In order to remove the effect of Fermi cut-off, the curves in **a** are symmetrized about the Fermi level, and the ones in **b** are divided by the Fermi function at 150K. **c**, The Fermi surface. The $\mathbf{k}_\mathrm{F}$ points studied in Fig.5a and Supplementary Figure **14** are marked by colored circles. **d**, The peak width of spectra (or Γ_single) in **a** and **b**. The orange arrows in **c** and **d** indicate the momentum region where the gapless Fermi arc was previously claimed to emerge at T_c. The almost isotropic Γ_single is obtained in this momentum region.

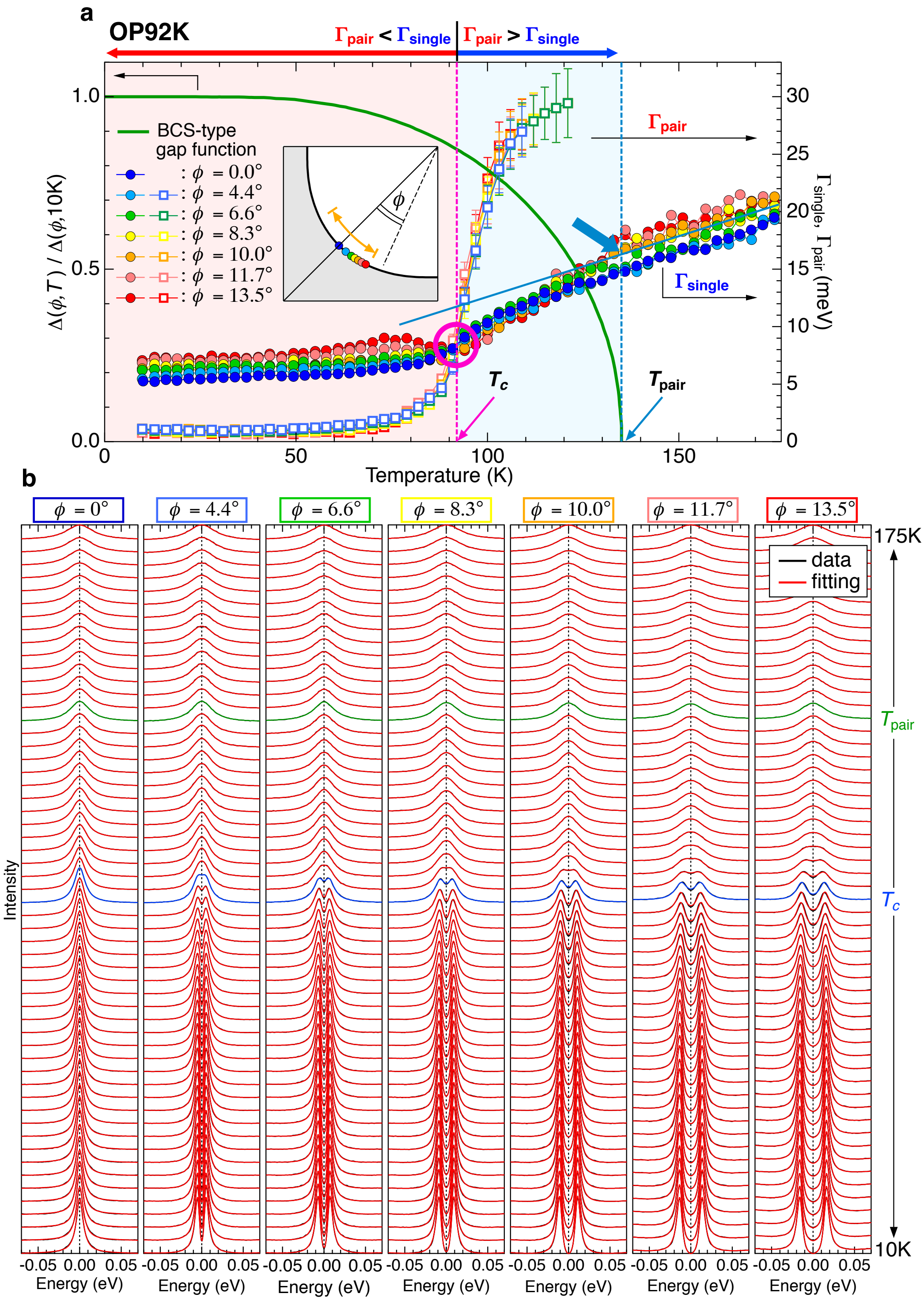

Supplementary Figure 14: Fitting results for OP92K data with the model function of Eq.(S1). **a**, Same panel as Fig. 5a in the main paper: single-particle scattering rate ($\Gamma_{\rm single}$) and pair breaking rate ($\Gamma_{\rm pair}$) extracted by setting the energy gap (Δ) to be the BCS-type gap function (a green curve) with the onset of 135K. **b**, ARPES spectra (black curves) and fitting results (red curves) are overlapped. Fermi angle (ϕ) is described in the top of each panel, and the corresponding $\mathbf{k}_{\rm F}$ point is marked with a circle in the inset of **a**. We added a small background linear in energy ($\propto |\omega|$) to the fitting function $A(k_F, \omega)$ for $\phi = 11.7°$ and $13.5°$, in order to properly extract the scattering rates.

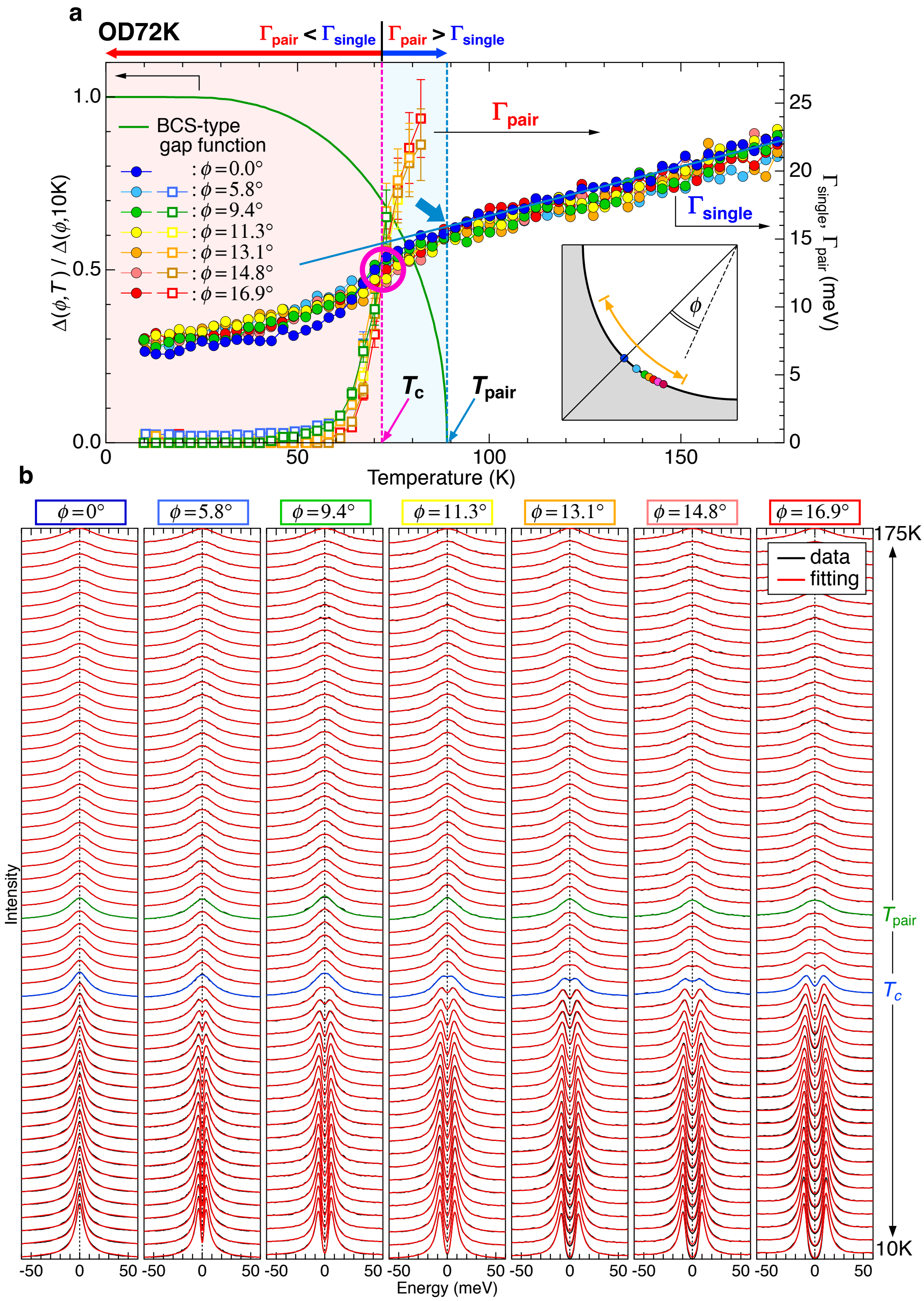

Supplementary Figure 15: Fitting results for OD72K data with the model function of Eq.(S1). **a**, Same panel as Fig. 5b in the main paper: single-particle scattering rate ($\Gamma_{\rm single}$) and pair breaking rate ($\Gamma_{\rm pair}$) extracted by setting the energy gap (Δ) to be the BCS-type gap function (a green curve) with the onset of 89K. **b**, ARPES spectra (black curves) and fitting results (red curves) are overlapped. Fermi angle (ϕ) is described in the top of each panel, and the corresponding $\mathbf{k}_{\rm F}$ point is marked with a circle in the inset of **a**. We added a small background linear in energy ($\propto |\omega|$) to the fitting function $A(k_F, \omega)$ for $\phi = 14.8^\circ$ and 16.9°, in order to properly extract the scattering rates.

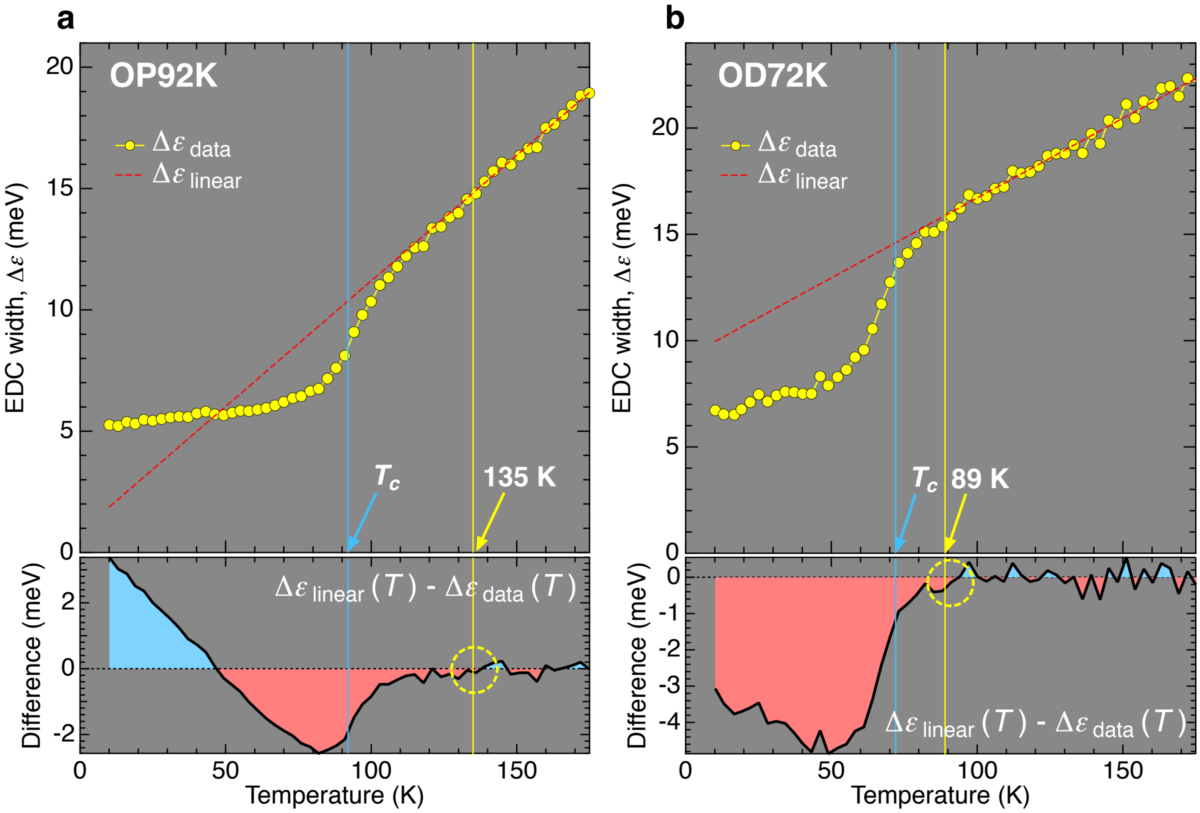

Supplementary Figure 16: Signature of the pairing detected in the nodal spectra. Temperature evolution of peak width ($\Delta\varepsilon_{\rm data}$) of the symmetrized EDCs for OP92K (**a**) and OD72K (**b**) are plotted. In the bottom of each panel, the deviation of the $\Delta\varepsilon_{\rm data}(T)$ from the T-linear behaviors (red dashed lines) are estimated. The onset temperatures of the deviation are marked by yellow circles. The $T_{\rm c}$ and $T_{\rm pair}$ determined in the main paper are indicated by a light-blue and yellow arrow, respectively.

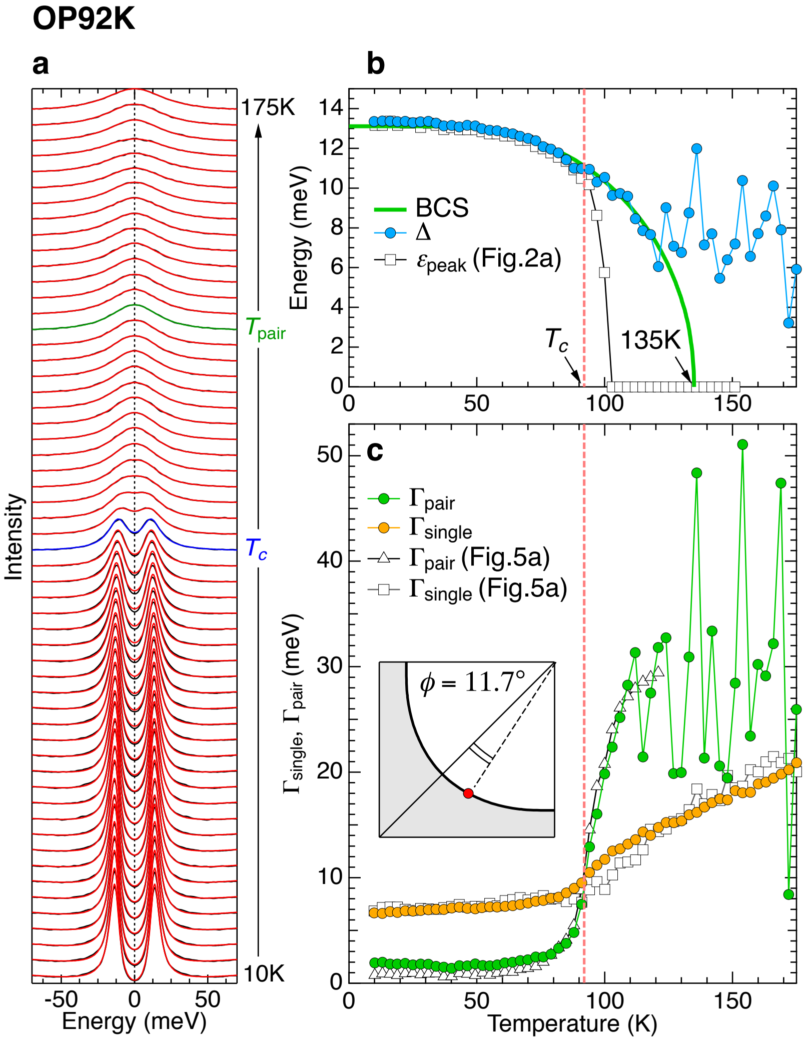

Supplementary Figure 17: Fitting results with a different scheme from that used in the main paper; here we input model values to $\Gamma_{\rm single}(T)$ in Eq.(S1) and extract the other two parameters of $\Delta(T)$ and $\Gamma_{\rm pair}(T)$. **a**, The off-nodal ARPES spectra of OP92K (black curves) and the fitting results (red curves) at $\phi = 11.7^\circ$ (red circle in the inset of **c**). We used the nodal $\Gamma_{\rm single}(T)$ slightly shifted in the magnitude (orange circles in **c**), and added a small background linear in energy ($\propto |\omega|$) to the $A(k_F, \omega)$ in Eq.(S1) for a realistic fitting to extract the other two parameters (Δ and $\Gamma_{\rm pair}$). **b**, The obtained $\Delta(T)$ are plotted (light blue circles). For comparison, the BCS-type gap function with the onset of 135K (green curve) and the peak energies of symmetrized EDCs ($\varepsilon_{\rm peak}$s in Fig.2a of the main paper) are also shown. **c**, The used $\Gamma_{\rm single}(T)$ for the fitting analysis (orange circles) and the extracted $\Gamma_{\rm pair}(T)$ (green circles) are plotted. For comparison, the $\Gamma_{\rm single}(T)$ (open black squares) and $\Gamma_{\rm pair}(T)$ (open black triangles) obtained in the main paper with a different fitting scheme (Fig.5a of the main paper) are also shown.

Supplementary Note 1: Samples and Experimental method

Optimally doped $Bi_2Sr_2CaCu_2O_{8+\delta}$ (OP92K) and overdoped $(Bi,Pb)_2Sr_2CaCu_2O_{8+\delta}$ (OD72K) single crystals with T_c=92K and 72K, respectively, were grown by the conventional floating-zone (FZ) technique. Magnetic susceptibilities for these single crystals are shown in Supplementary Figure **1**. Sharp superconducting transitions with ∼1 K (OP92K) and ∼3 K (OD72K), indicative of a high quality, are confirmed. ARPES data were accumulated using a laboratory-based system consisting of a Scienta R4000 electron analyzer and a 6.994 eV laser (the 6th harmonic of Nd:YVO4 quasi-continuous wave with a repetition rate of 240 MHz). The overall energy resolution in the ARPES experiment was set to 1.4 meV for all the measurements.

In order to accomplish the temperature scan of spectra at a high precision, we made a special sample stage; we use a heat switch to thermally isolate the sample holder with a heater and a temperature sensor (totally $20 \times 30 \times 10$ mm size) from the rest of the system when sweeping the sample temperature. With this technique, the samples are efficiently heated up from 10K up to 300K while keeping the temperature of manipulator rod other than the sample stage lower than 35K. It minimizes the degassing and keeps the pressure in the measurement chamber better than 2×10^{-11} torr during the entire temperature sweeping. The method of local heating also prevents the thermal expansion of the *long* manipulator rod, and thus the sample position becomes unchanged with temperature. It enabled us to take data in fine temperature steps with the automated measurement of temperature scan from precisely the same spot on the crystal surface, which were essential to achieve the aim of this study.

In Supplementary Figs. **2** and **3**, we show the ARPES spectra of OP92K obtained at various $\mathbf{k}_F$ points with sweeping the temperature, with and without an offset, respectively. These are the original data used for the analysis in the main paper. The ARPES technique is sensitive to the condition of sample surface, and the biggest challenge in it is to prevent the aging of sample surface with time. In Supplementary Figure **4**, we carefully check the stability of sample surface in our experiment by comparing two sets of spectra measured before and after the temperature scan for all the momentum cuts we measured. While it took about 12 hours to complete each temperature sweeping, almost perfect reproducibility of spectral line shapes is confirmed for all the data. This ensures that our data measured with the new heating technique are reliable and suitable for conducing the detailed analysis presented in the paper. Supplementary Figure **5** shows the data set similar to those in Supplementary Figure **3**, but measured for OD72K.

Supplementary Note 2: Further Evidence for absence of the Fermi arc at T_c

In the main paper, we demonstrate that the *d*-wave gap with a point node persists at T_c by plotting the symmetrized EDCs at various $\mathbf{k}_F$ points around the node (see Fig. 3d). Here we present a further evidence that the conclusion is robust and it is not sensitive to the selection of $\mathbf{k}_F$ value. Supplementary Figure **6**a shows the Fermi-function divided band dispersions (same as Fig.3a in the main paper) measured at T_c along several momentum cuts (color lines in Supplementary Figure **6**c). The corresponding EDCs are plotted in Supplementary Figure **6**b. Along the diagonal cut (the most left panel), the spectra with one peak crosse E_F due to the gap node. In contrast, two peak structure (marked with arrows), signifying a gap existence, is observed at $\mathbf{k}_F$ (painted with red) for the other directions. This is a direct evidence that the *d*-wave gap with a point node persists, thus the Fermi arc is absent at T_c.

Supplementary Note 3: Temperature evolution of the "artificial" Fermi arc

The Fermi arc [1] is one of the most interested features in cuprates. Previous studies have proposed that the gapless Fermi arc abruptly emerges at T_c, and expands linearly with a further increase of temperature [2–4]. The arc length was claimed to be extrapolated to zero at $T = 0$ [3, 4]. The so-called "nodal liquid"

behavior has been having a huge influence on the condensed matter community.

In the main paper, we argue that the tracking of peak positions of the symmetrized EDCs ($\varepsilon_{\rm peak}$s) underestimates the gap closing temperature, thus provides an "artificial" Fermi arc. Here we demonstrate that even such an artificial arc behaves quite differently from the previous expectation, when high resolution data by a laser ARPES are examined. In Supplementary Figure **7**a and **7**a, we estimate the length of arc with $\varepsilon_{\rm peak}$=0 as a function of temperature for OP92K and OD72K, respectively. In the same panels, the nodal liquid behaviors are superimposed. Our results are significantly off these lines. As discussed in the main paper, the spectral line-shape is determined not only by the energy gap (Δ), but also by the mutual relation between the single-particle scattering rate ($\Gamma_{\rm single}$) and the pair breaking rate ($\Gamma_{\rm pair}$). Therefore, it is rather reasonable that the growth of arc with $\varepsilon_{\rm peak}$=0 becomes arbitrary.

Supplementary Note 4: Effect of scattering rates, $\Gamma_{\rm single}$ and $\Gamma_{\rm pair}$, on the spectral line shape

In the analysis, we used the spectral function with a phenomenological self-energy (ref. [5]) given by

$$A(\mathbf{k},\omega) = \frac{1}{\pi}\cdot\frac{\Sigma''(\mathbf{k},\omega)}{[\omega-\varepsilon(\mathbf{k})-\Sigma'(\mathbf{k},\omega)]^2+\Sigma''(\mathbf{k},\omega)^2},$$

$$\Sigma(\mathbf{k},\omega) = -i\Gamma_{\rm single} + \Delta^2/[\omega+\varepsilon(\mathbf{k})+i\Gamma_{\rm pair}]. \tag{S1}$$

Here Δ is the energy gap, and $\varepsilon(k)$ the energy dispersion with $\varepsilon(\mathbf{k}_{\rm F})$ =0. $\Gamma_{\rm single}$ and $\Gamma_{\rm pair}$ are the single particle scattering rate and the pair breaking rate, respectively. In Supplementary Figure **8**, we examine the effect of these scattering rates on the spectral line shape at $\mathbf{k}_{\rm F}$ with fixing Δ to 10 meV. To make it realistic, all the curves plotted are convoluted with a Gaussian that has the width of the experimental energy resolution ($\Delta\varepsilon$ = 1.4meV). Nevertheless, note that the $\Delta\varepsilon$ value is so small that the difference in shape from original curves is negligible. Figure **8**a shows the $\Gamma_{\rm single}$ dependence of the spectral function when the $\Gamma_{\rm pair}$ is fixed to zero. One finds that the intensity at $E_{\rm F}$ is always zero regardless of the $\Gamma_{\rm single}$ value, whereas the spectral width gets broadened with increasing $\Gamma_{\rm single}$. This situation strongly contrasts to that in our ARPES data (Supplementary Figure **2**), which clearly show the filling of spectral weight at $E_{\rm F}$ with increasing temperature. The behavior of gap filling can be produced by inputting non-zero $\Gamma_{\rm pair}$ values in Eq.(S1). It is demonstrated in Supplementary Figure **8**b, where the spectra with several $\Gamma_{\rm pair}$ values are calculated with fixing $\Gamma_{\rm single}$ to zero. Importantly, the energy position of spectral peak ($\varepsilon_{\rm peak}$) dramatically decreases with an increase of the $\Gamma_{\rm pair}$ value as a consequence of the spectral filling around $E_{\rm F}$, and eventually it becomes zero. This simulation indicates that the peak energy underestimates the "real" energy gap ($\varepsilon_{\rm peak} < \Delta$) when the magnitude of $\Gamma_{\rm pair}$ becomes large.

Here we should point out that the following self-energy has been used to estimate the magnitude of Δ by a fitting to the ARPRS spectra in the many previous reports,

$$\Sigma(\mathbf{k}_{\rm F},\omega) \equiv -i\Gamma_{\rm single} + \Delta^2/\omega. \tag{S2}$$

This is the same formula as Eq.(S1) setting $\Gamma_{\rm pair}$ = 0, and thus it always provides one the spectral intensity of zero at $E_{\rm F}$ as discussed above (see Supplementary Figure **8**a). We emphasize that the spectral weight around $E_{\rm F}$ observed in the ARPES data was previously attributed to the spectral broadening coming from the finite energy resolution in ARPES (for example, see the supplementary information of ref.[2]). The extremely high energy resolution in our equipment enabled us to reveal that the spectral weight at $E_{\rm F}$ is not an artifact due to the experimental resolution, but an intrinsic signature of the pair breaking effect. This signifies that the Eq.(S1) is more suitable than Eq.(S2) as the model spectral function of cuprates, and that the effect of $\Gamma_{\rm pair}$ must be taken into account to properly estimate the value of Δ from the spectral shape.

In Supplementary Figure **8**c, we simulate a special case setting $\Gamma_{\rm pair}$ = $\Gamma_{\rm single}$ in Eq.(S1). The self-

energy and the corresponding spectral function at $\mathbf{k}_\mathrm{F}$ under this condition are derived as follow;

$$\Sigma(\mathbf{k}_\mathrm{F},\omega) = -i\Gamma + \Delta^2/[\omega + i\Gamma],$$

$$A(\mathbf{k}_\mathrm{F},\omega) = \frac{1}{2}\left[\frac{\Gamma}{(\omega-\Delta)^2+\Gamma^2} + \frac{\Gamma}{(\omega+\Delta)^2+\Gamma^2}\right], \tag{S3}$$

where $\Gamma \equiv \Gamma_\mathrm{single} = \Gamma_\mathrm{pair}$. This formula, consisting of double Lorentzian functions, is familiar as the phenomenological BCS spectral function [6–8]. The calculated spectra for various Γ values are plotted in Supplementary Figure **8**c. One can confirm a couple of discrepancies in these from our data. First, the spectral peak in the ARPRS data has an asymmetric shape about the peak energy, differently from the Lorentzian shape. Secondly, the effect of gap filling in this simulation is quite sensitive to the magnitude of Γ, and it is far more drastic than that observed in our data at elevated temperatures. These lead us to conclude that the Eq.(S3) is not a proper formula of spectral function for cuprates either; that is, the assumption of $\Gamma_\mathrm{pair} = \Gamma_\mathrm{single}$ is not suitable.

We also stress that the Dynes function [9] given by,

$$I_\mathrm{DOS}(\omega) = \mathrm{Re}\left[\frac{\omega - i\Gamma}{\sqrt{(\omega-i\Gamma)^2-\Delta^2}}\right], \tag{S4}$$

is the density-of-states version of Eq.(S3) with $\Gamma \equiv \Gamma_\mathrm{single} = \Gamma_\mathrm{pair}$, hence it is also not adequate for cuprates. This is understandable because there is no good physical reason that these two different scattering rates should be equal. Our results demonstrate that a proper combination of Γ_single and Γ_pair is required to reproduce the data.

In the previous reports [10–12], the spectrum integrated along a selected momentum cut was utilized to study the gap evolution with temperature and its relation with the electron scattering. Here we argue the necessity of investigating the one-particle spectrum, rather than its momentum integration, for such a study. In Supplementary Figure **9**, we demonstrate that a momentum-integrated spectrum, $I_\mathrm{DOS}(\omega) = \int A(\mathbf{k},\omega)d\mathbf{k}$ for $A(\mathbf{k},\omega)$ of Eq.(S1), is equally sensitive to the Γ_single value (Supplementary Figs. **9**a and **9**b) and the Γ_pair value (Supplementary Figs. **9**c and **9**d); these two scattering rates both fill the spectral intensity around E_F, while the gap size Δ is fixed to a constant value (15 meV). The spectra of $I_\mathrm{DOS}(\omega)$ calculated under the condition of $\Gamma_\mathrm{single} = \Gamma_\mathrm{pair}$ (Supplementary Figs. **9**e and **9**f) reproduce those in Supplementary Figs. **9**a and **9**b. As naturally expected, only the half values of Γ_single and Γ_pair are sufficient to cause the same effect in this case. Notably, the obtained curves are identical to Dynes function in Eq.(S4) as pointed out above, which is demonstrated for several values of Γ in Supplementary Figure **9**g. These simulations providing identical results (Supplementary Figs. **9**a, **9**c, **9**e, and **9**g) signify that the momentum-integrated spectrum is not capable of extracting the mutual relationship between the two scattering rates (Γ_single and Γ_pair).

In contrast to it, the effects of Γ_single and Γ_pair on the line shape of the momentum-resolved spectrum $A(\mathbf{k},\omega)$ are independent, broadening the width and filling the weight around E_F, respectively. This circumstance is demonstrated in Supplementary Figure **8**, and also seen in Supplementary Figure **9h**, at which the spectra at $\mathbf{k}_\mathrm{F}$ are extracted from images in Supplementary Figs. **9b**, **9d**, and **9f**. (Spectral colors of red, blue, and green are corresponding to those of thick dashed lines on the images.) Therefore, the precise investigation for the line shape of one-particle spectra is required to separate the three important parameters (Γ_single, Γ_pair, and Δ) in Eq.(S1). The main challenge in this task, which has been troubling the ARPES people, is to accomplish both of a sufficient energy resolution and an ultra-high statistics in the ARPES spectra with no noise at a time, which are usually conflicting with each other. We have overcome this difficulty by using a laser ARPES with a unique cold finger (mentioned in "Experimental Method"), which was constructed in our lab. We emphasize that the Eq.(S1) is a minimal model to reproduce the ARPES data, thus the relation among the three physical parameters, revealed in the present work, provides a key ingredient to formulate the mechanism of the high-T_c superconductivity

in cuprates.

Supplementary Note 5: Signature of a gap existence in the off-nodal, single-peak spectra above T_c

Here we demonstrate that the off-nodal spectra above T_c exhibit a signature of the gap opening even after becoming one-peak structure. The bottom panels of Supplementary Figure **10**a and **10**b plot the symmetrized EDCs of OP92K above T_c measured at the node ($\phi = 0°$) and off the node ($\phi = 13.5°$), respectively. Two peaks seen in the off-nodal spectra merge to a single peak at T_{one}=103K (Supplementary Figure **10**a). For clarity, the spectra above the T_{one} are indicated with rainbow colors, whereas those at the lower temperatures with gray colors in the both of Supplementary Figure **10**a and **10**a.

We find that the single-peak spectra off the node (rainbow curves in Supplementary Figure **10**a) become sharper with increasing temperature up to $\sim$ 135K (T_{pair}) before eventually broadened at higher temperatures. The anomalous feature is more clearly demonstrated in the upper panel of Supplementary Figure **10**a, where the spectra below and above the T_{pair} are separated with an offset. As seen in the the magnified insets, the spectral tail shifts to lower energies in the range of $T < T_{pair}$ (a magenta arrow), while it to the higher energies in $T > T_{pair}$ (a black arrow). It strongly contrasts to the case at the node (Supplementary Figure **10**b); the spectral tail keeps shifting toward higher energies at elevated temperatures in the both temperature ranges (black arrows). We summarize these results in Supplementary Figure **10**c, by plotting the spectral width $\Delta\varepsilon$ (HWHM) at and off the node as a function of temperature. The anomalous decrease of $\Delta\varepsilon$ is seen only for the data off the node (orange circles). As discussed in the main paper, a plausible explanation for it is that the off-nodal spectra have small energy gaps, even though they exhibit single peak shapes, and they become sharper up to the temperate (T_{pair}) at which the energy gap completely closes.

Supplementary Note 6: Spectral fitting under the assumption of $\Delta(T) \equiv \varepsilon_{\mathbf{peak}}(T)$

In the main paper, we claim that the energy of spectral peak underestimates the energy gap ($\varepsilon_{peak} < \Delta$) close to the node at high temperatures, at which the spectral peak width becomes larger than the magnitude of the energy gap. Here we perform a spectral fitting to our ARPES data with Eq.(S1) [same as Eq.(1) in the main paper], assuming that these two values are identical [$\Delta(T) \equiv \varepsilon_{peak}(T)$, upper panel of Supplementary Figure **11**a (OP92K) and Supplementary Figure **12**a (OD72K)], and demonstrate that the extracted parameter has an unrealistic behavior, reflecting the irrelevance of $\Delta(T) \equiv \varepsilon_{peak}(T)$. The middle and bottom panels of Supplementary Figure **11**a plot the $\Gamma_{single}(T)$ and $\Gamma_{pair}(T)$ of OP92K, respectively, obtained by the fitting to the spectra in the momentum region, where the Fermi arc was previously claimed to appear at T_c (orange arrow in the insets of Supplementary Figure **11**b). For a comparison, we also plot in Supplementary Figure **11**b (OP92K) the corresponding results for $\Delta(T)$ of the BCS-like gap function, which are presented in the main paper (Fig. 5a). The difference between the two fitting-results is very clear; the former (Supplementary Figure **11**a) shows an abnormal upturn in the $\Gamma_{single}(T)$ curves on cooling in the gapped momentum region, and it contrasts to the latter result (Supplementary Figure **11**b) showing almost identical curves in $\Gamma_{single}(T)$ for all ϕs. In Supplementary Figure **12**, we present the same data set as in Supplementary Figure **11**, but for OD72K. Similarly to the case of OP92K, an abnormal upturn is obtained in the $\Gamma_{single}(T)$ when $\Delta(T) \equiv \varepsilon_{peak}(T)$ (the middle panel of Supplementary Figure **12**a), and it is corrected by setting $\Delta(T)$ to be the BCS-like gap function (the middle panel of Supplementary Figure **12**b).

The behavior of the abnormal upturn is interpreted as follows. An energy gap is still open above T_c even though the spectrum has a single peak, and thus the spectral peak width overestimates the scattering rate. Since the degree of the overestimation gets higher with an increase of the energy gap on cooling, the upturn in the $\Gamma_{single}(T)$ appears. The upturn seems to be more enhanced with getting away from the node (see middle panel of Supplementary Figure **11**a and Supplementary Figure **12**a). This is

also expected as the overestimation becomes more serious toward the antinode with a larger energy gap. This scenario is further validated by the fact that the upturns start around $T \sim 130$K and $T \sim 90$K for OP92K and OD72K, respectively, which are consistent with the onset temperatures of pair formation determined in the main paper.

One might think that the cuprates are known to have a significantly anisotropic scattering mechanism, thus the situation in Supplementary Figure **11**a (OP92K) and Supplementary Figure **12**a (OD72K) with the strong momentum variation could be intrinsic. To argue against this, here we demonstrate that even the optimally doped Bi2212, in fact, has an isotropic scattering mechanism around the node. Supplementary Figures **13**a and **13**b show the spectra at various $\mathbf{k}_\mathrm{F}$ points over a wide ϕ angle measured at the lowest temperature (T=10K) and above the pairing temperature (T=150K), respectively. At these temperatures, the value of Γ_single can be estimated simply from the peak width of spectra, since the magnitude of Γ_pair is negligible at 10K, and the second term in Eq.(S1) with Γ_pair is irrelevant above the pairing temperature. The values of Γ_single estimated from the peak width of spectra in Supplementary Figure **13**a and **13**b are plotted in Supplementary Figure **13**d with blue and red circles, respectively. The magnitude is almost constant over a wide ϕ centered at the node for both the temperatures, which indicates that the scattering mechanism near the node is isotropic. This strongly supports our assertion that the anisotropic behavior of Γ_single with an upturn on cooling is an artifact. Beyond $|\phi| \approx 15°$, the scattering rate abruptly increases. This signifies the evolution of the competing pseudogap, which becomes dominant around the antinode [13–16].

Supplementary Note 7: Fitting curves reproducing the ARPES spectra

In the main paper, we demonstrate the spectral fitting with a minimal model of Eq.(S1) to the data of OP92K at $\phi = 13.5°$ (Fig.5c), as a typical example. Here we present all of the fitting results for the ARPES spectra of OP92K and OD72K, which provide the physical parameters plotted in Fig. 5a (Supplementary Figure **14**a) and Fig. 5b (Supplementary Figure **15**a), respectively. Supplementary Figure **14**b and **15**b shows the ARPES spectra (black curves) and fitting results (red curves) for OP92K and OD72K, respectively. The data are almost perfectly reproduced by the fitting curves.

Supplementary Note 8: The pairing signature detected in the nodal $\Gamma_\mathrm{single}(T)$

The single-particle scattering rate (Γ_single) at the node is extracted simply from the spectral width, thus it can be precisely determined without any models. Supplementary Figure **16**a and **16**b plots the temperature dependence of EDC width $\Delta\varepsilon$ at the nodal $\mathbf{k}_\mathrm{F}$ point of OP92K and OD72K, respectively (same as $\Gamma_\mathrm{single}(T)$ at $\phi = 0°$ in Figs. 5a and 5b). We find that the curves of $\Delta\varepsilon(T)$ deviate from the T-linear behavior ($\Delta\varepsilon_\mathrm{linear}(T)$) in the both samples. It is demonstrated more clearly in the bottom panels by plotting the amount of divination $[\Delta\varepsilon_\mathrm{linear}(T) - \Delta\varepsilon_\mathrm{data}(T)]$. The onset temperatures of the deviation are estimated to be ~ 135K and ~ 89K for OP92K and OD72K, respectively (yellow circles). The suppression of the scattering rate should be tied to the electron pairing. We found that the onset temperatures are indeed coincident with the T_pair values (gap opening temperatures) estimated from the off-nodal spectra in the main paper.

Supplementary Note 9: Fitting results with a different scheme

The model spectral function of Eq.(S1) [same as Eq.(1) in the main paper] has three parameters of the energy gap (Δ), the single-particle scattering rate (Γ_single), and the pair breaking rate (Γ_pair). In the main paper, we assume the Δ to be BCS-type gap function with the onset temperature much higher than T_c, and extract the other two parameters of Γ_single and Γ_pair by fitting the model function to the data. Here we perform a new analysis with a different fitting scheme to justify the BCS-type gap

function assumed. In the new analysis, we use the nodal $\Gamma_{\mathrm{single}}(T)$, which can be determined without models, for the fitting of the off-nodal spectra, and set the other two parameters of Δ and Γ_{pair} free. The underlying idea for it is that the scattering mechanism is rather isotropic near the node. To make the fitting realistic, we slightly shifted the nodal $\Gamma_{\mathrm{single}}(T)$ in the magnitude. The fitting results (OP92K, $\phi = 11.7°$) are demonstrated in Supplementary Figure **17**a. The fitting spectra (red curves) reproduce the data (black curves) reasonably well. The extracted Δ and Γ_{pair} also more or less agree to the results presented in the main paper, while these values above $\sim$ 120K are not properly extracted owing to the small magnitudes of Δ. This result justifies that the $\Delta(T)$ in cuprates has the BCS-type gap function with an onset higher than T_c, and validates the requirement of pair breaking rate (Γ_{pair}) to reproduce the ARPES spectra.

Supplementary References

[1] Norman, M. R. *et al.* Destruction of the Fermi surface in underdoped high-T_c superconductors. *Nature* **392**, 157–160 (1998).

[2] Lee, W. S. *et al.* Abrupt onset of a second energy gap at the superconducting transition of underdoped Bi2212. *Nature* **450**, 81–84 (2007).

[3] Kanigel, A. *et al.* Evolution of the pseudogap from Fermi arcs to the nodal liquid. *Nat. Phys.* **2**, 447–451 (2006).

[4] Nakayama, K. *et al.* Evolution of a Pairing-Induced Pseudogap from the Superconducting Gap of $(Bi,Pb)_2Sr_2CuO_6$. *Phys. Rev. Lett.* **102**, 227006 (2009).

[5] Norman, M. R., Randeria, M., Ding, H., and Campuzano, J. C. Phenomenology of the low-energy spectral function in high-T_c superconductors. *Phys. Rev. B* **57**, R11093 (1998).

[6] Schrieffer, J. R. Theory of Superconductivity. *(W. A. Benjamin, New York, 1964), p. 122.*

[7] Campuzano, J. C. *et al.* Direct observation of particle-hole mixing in the superconducting state by angle-resolved photoemission. *Phys. Rev. B* **53**, R14737–R14740 (1996).

[8] Matsui, H. *et al.* BCS-Like Bogoliubov Quasiparticles in High-T_c Superconductors Observed by Angle-Resolved Photoemission Spectroscopy. *Phys. Rev. Lett.* **90**, 217002 (2003).

[9] Dynes, R. C., Narayanamurti, V., and Garno, J. P. Direct measurement of quasiparticle-lifetime broadening in a strong-coupled superconductor. *Phys. Rev. Lett.* **41**, 1509–1512 (1978).

[10] Reber, T. J. *et al.* The origin and non-quasiparticle nature of Fermi arcs in $Bi_2Sr_2CaCu_2O_{8+\delta}$. *Nat. Phys.* **8**, 606–610 (2012).

[11] Reber, T. J. *et al.* Prepairing and the "filling" gap in the cuprates from the tomographic density of states. *Phys. Rev. B* **87**, 060506 (2013).

[12] Kondo, T. *et al.* Formation of Gapless Fermi Arcs and Fingerprints of Order in the Pseudogap State of Cuprate Superconductors. *Phys. Rev. Lett.* **111**, 157003 (2013).

[13] Kondo, T., Khasanov, R., Takeuchi, T., Schmalian, J., and Kaminski, A. Competition between the pseudogap and superconductivity in the high-T_c copper oxides. *Nature* **457**, 296–300 (2009).

[14] Kondo, T. *et al.* Disentangling Cooper-pair formation above the transition temperature from the pseudogap state in the cuprates. *Nat. Phys.* **7**, 21–25 (2010).

[15] Lee, J. *et al.* Spectroscopic fingerprint of phase-incoherent superconductivity in the underdoped $Bi_2Sr_2CaCu_2O_{8+\delta}$. *Science* **325**, 1099–1103 (2009).

[16] Kohsaka, Y. *et al.* How Cooper pairs vanish approaching the Mott insulator in $Bi_2Sr_2CaCu_2O_{8+\delta}$. *Nature* **454**, 1072–1078 (2008).
